# Identification of factors associated with duplicate rate in ChIP-seq data

**DOI:** 10.1371/journal.pone.0214723

**Published:** 2019-04-03

**Authors:** Shulan Tian, Shuxia Peng, Michael Kalmbach, Krutika S. Gaonkar, Aditya Bhagwate, Wei Ding, Jeanette Eckel-Passow, Huihuang Yan, Susan L. Slager

**Affiliations:** 1 Division of Biomedical Statistics and Informatics, Department of Health Sciences Research, Mayo Clinic, Rochester, Minnesota, United States of America; 2 Division of Research and Education Support Systems, Department of Information Technology, Mayo Clinic, Rochester, Minnesota, United States of America; 3 Division of Hematology, Mayo Clinic, Rochester, Minnesota, United States of America; Universidad Nacional Autonoma de Mexico, MEXICO

## Abstract

Chromatin immunoprecipitation and sequencing (ChIP-seq) has been widely used to map DNA-binding proteins, histone proteins and their modifications. ChIP-seq data contains redundant reads termed duplicates, referring to those mapping to the same genomic location and strand. There are two main sources of duplicates: polymerase chain reaction (PCR) duplicates and natural duplicates. Unlike natural duplicates that represent true signals from sequencing of independent DNA templates, PCR duplicates are artifacts originating from sequencing of identical copies amplified from the same DNA template. In analysis, duplicates are removed from peak calling and signal quantification. Nevertheless, a significant portion of the duplicates is believed to represent true signals. Obviously, removing all duplicates will underestimate the signal level in peaks and impact the identification of signal changes across samples. Therefore, an in-depth evaluation of the impact from duplicate removal is needed. Using eight public ChIP-seq datasets from three narrow-peak and two broad-peak marks, we tried to understand the distribution of duplicates in the genome, the extent by which duplicate removal impacts peak calling and signal estimation, and the factors associated with duplicate level in peaks. The three PCR-free histone H3 lysine 4 trimethylation (H3K4me3) ChIP-seq data had about 40% duplicates and 97% of them were within peaks. For the other datasets generated with PCR amplification of ChIP DNA, as expected, the narrow-peak marks have a much higher proportion of duplicates than the broad-peak marks. We found that duplicates are enriched in peaks and largely represent true signals, more conspicuous in those with high confidence. Furthermore, duplicate level in peaks is strongly correlated with the target enrichment level estimated using nonredundant reads, which provides the basis to properly allocate duplicates between noise and signal. Our analysis supports the feasibility of retaining the portion of signal duplicates into downstream analysis, thus alleviating the limitation of complete deduplication.

## Introduction

Chromatin immunoprecipitation (ChIP) and sequencing (ChIP-seq) has been widely used for genome-wide mapping of transcription factors, chromatin regulators and histone modifications [1]. ChIP-seq data contain redundant reads (duplicates), which are reads or pairs of reads having identical or near-identical (due to sequencing errors) sequences and mapping to the same genomic position and strand [2–4]. Duplicate rate reflects library complexity, which is an important ChIP-seq quality metric representing the nonredundant fraction (NRF) of uniquely mapped reads (i.e., NRF = number of positions / total uniquely mapped reads) [5, 6]. It is recommended that NRF should reach approximately 0.8 (i.e., 20% or less duplicates) for narrow-peak marks with 10 million and for broad-peak marks with 20 million uniquely mapped reads [5].

During library preparation, ChIP DNA needs to be polymerase chain reaction (PCR) amplified. This step introduces bias, as some of the templates are amplified more efficiently than the others, leading to the sequencing of identical copies from the same DNA fragment [7]. PCR amplification is a major source of redundant reads, the so-called “PCR duplicates” [3, 8]. PCR duplicates are more abundant when the library is deeply sequenced [5], or constructed from insufficient DNA molecules due to low immunoprecipitation (IP) efficiency [9] or little starting material (such as single cells) [10, 11]; in the latter cases, more PCR cycles are often required to generate sufficient DNA for sequencing. PCR duplicates are artifacts that need to be filtered out. In addition, a small proportion of duplicates may be caused by erroneous mapping of reads actually originating from regions of segmental duplication. Another major type of duplicates is “natural duplicates”, which represent true signals [8]. Natural duplicates arise from sequencing of independent DNA fragments derived from the same genomic locations [6]. The differentiation of PCR versus natural duplicates is important but computationally challenging.

Duplicates could be identified using *de novo*- and alignment-based approaches. Several *de novo*-based methods have been developed to identify duplicates directly from raw Illumina sequencing data, such as FastUniq [3] and Fastx-Toolkit Collapser (http://hannonlab.cshl.edu/fastx_toolkit/) for identical duplicates and CD-HIT-DUP [12], Fulcrum [13] and GPU-DupRemoval [14] for near-identical duplicates. More often, duplicates are identified from coordinate-sorted alignments, using tools such as SAMtools markdup command [15] and Picard MarkDuplicates command (http://broadinstitute.github.io/picard/). For both approaches, duplicate rate is overestimated for single-end compared to paired-end reads [2]. For paired-end reads, only those with the same mapping location and strand of both ends are counted as duplicates; for single-end reads, however, some of the reads with the same mapping location and strand, which are classified as duplicates, may actually come from fragments of different sizes.

To separate PCR and natural duplicates, methods were developed to use unique molecular identifiers (UMIs, they are random oligonucleotide barcodes) to tag individual DNA fragments during library preparation [16]. After sequencing, natural duplicates could be confidently separated from PCR duplicates, since the former are unlikely to share the same UMI but the latter should [8, 10, 16]. Though showing evidence of improved accuracy in variant discovery and gene expression quantification [8], the UMI-based methods are not routinely used [7].

It is a common practice to filter out duplicates in ChIP-seq data [5, 6, 17], which has been shown to improve specificity of peak calling by Model-based analysis of ChIP-Seq (MACS) [17] without a noticeable loss of sensitivity [2]. However, excluding duplicates has the side effect of underestimating the read coverage in peaks [4]. While MACS provides the options to keep a pre-defined number of reads per position or to calculate the maximum number of reads to keep based on the binomal distribution (https://github.com/taoliu/MACS), both options treat the peak and non-peak regions in the same way.

It is already known that the distribution of duplicates is far from random in the genome, at least for RNA-seq [10] and ChIP-seq data [2]. Natural duplicates are much more abundant in highly expressed genes [10]. For example, using RNA-seq data from individuals in the 1000 Genomes Project, it was estimated that only 5–30% of the redundant reads were PCR duplicates and over 70% represented natural duplicates in highly expressed genes [8]. Another study used UMIs to differentiate the two types of duplicates in RNA-seq, revealing that complete deduplication without relying on the UMIs led to about one-fourth false negatives in the detection of differential expression for highly expressed genes [7]. Not surprisingly, gene duplicate rate is well correlated with the length-normalized read counts (i.e., reads per kilobase (kb)) in RNA-seq [10]. Analogous to the exons from expressed genes that together account for about 2% of the human genome, ChIP-seq peaks from narrow-peak marks only cover a small portion (approximately 1–2%) of the mappable human genome. Thus, the probability of sequencing identical but independent fragments (i.e., natural duplicates) is much higher in the peaks relative to the non-peak regions. We and others found that duplicates are particularly enriched in peaks from narrow-peak marks and reasoned that most of the duplicates within peaks should represent true signal [2, 18]. We also found that duplicate rates are much lower in broad peaks from histone H3 lysine 27 trimethylation [18], suggesting that duplicate removal should have less impact for broad-peak marks.

In this study, we further investigated the distribution of duplicates in ChIP-seq peaks versus non-peak regions, evaluated the impact of duplicate removal on peak calling and signal quantification, and identified the factors that are strongly associated with duplicate level. Using public ChIP-seq data from three narrow-peak marks, including estrogen receptor (ER) and nuclear respiratory factor 1 (NRF1) transcription factor and histone H3 lysine 4 trimethylation (H3K4me3), we found overrepresentation of duplicates in peaks, especially in those with high confidence. Duplicate level (redundant reads per kb) in peaks is highly dependent on the target enrichment level (nonredundant reads per kb), based on which we estimated that 51–62% of the duplicates in ER peaks and over 90% in NRF1 and H3K4me3 peaks are true signals. Broad-peak marks H3K27me3 and H3K36me3 had much lower duplicate rates in peaks than the above narrow-peak marks. A less obvious but similar trend of correlation was also observed between duplicate level and target enrichment for these two marks, and over 80% of the duplicates in peaks were predicted as signal. Thus, target enrichment level in peaks represents a reliable predictor of natural duplicates that should be included in the signal quantification.

## Materials and methods

### Test datasets

We downloaded eight public human ChIP-seq datasets (S1 Table). Four are from three narrow-peak marks, including 51 base pair (bp) single-end H3K4me3 data in HeLa cell line [19], 36-bp single-end data from ER in breast cancer cell lines [20], 50-bp single-end data from NRF1 in HepG2, K562 and MCF7 cell lines [21], as well as 101-bp paired-end data from H3K4me3 in lymphoblastoid cell lines [22]. The other four are from broad-peak marks H3K27me3 and H3K36me3, including 50- or 51-bp single-end data in fetal retinal tissue [23] and 36-bp single-end data in breast cancer cell lines [24]. For the ER dataset, the reads were originally aligned to the hg18 reference genome [20]. The binary alignment/map (BAM) files were downloaded from the National Center for Biotechnology Information (NCBI) Gene Expression Omnibus under the accession GSE32222. Sequences were extracted from the BAM files using the SamToFastq command from the Picard suite (http://broadinstitute.github.io/picard/). For the other datasets, the sequence read archive (SRA) files were downloaded from NCBI short reads archive and converted into FASTQ format using SRA toolkit (v2.5.4–1) (https://github.com/ncbi/sra-tools/). Only 1–50 bases were used for the H3K4me3 data in lymphoblastoid cell lines. The three H3K4me3 data in HeLa cell line were generated without PCR amplification of ChIP DNA, which represent an ideal source to examine the abundance and distribution of non-PCR duplicates.

### Read mapping and peak calling

Reads were aligned to the hg19 reference genome using Burrows-Wheeler Alignment tool (BWA) (v0.6.2 or v0.7.10) [25]. Only uniquely mapped reads with a minimum mapping quality score of 20 and no mismatch in the first five bp were used for further analysis. Alignments were position sorted using the SortSam command and duplicates were identified using the MarkDuplicates command from the Picard.

Peaks were called from BAM files both before and after duplicate removal. H3K27me3 and H3K36me3 peaks were called using spatial clustering approach for the identification of ChIP-enriched regions (SICER, v1.1), a program specifically developed for the identification of broad peaks [26], at the parameter settings “window size = 200, fragment size = 300, effective genome fraction = 0.75, gap size = 600 and FDR (false discovery rate) = 1E-2”. For ER, NRF1 and H3K4me3, peaks were identified using MACS (v2.0.10) [17], with the parameter settings "-f BAM -g hs—keep-dup all -q 0.01—nomodel". The empirical FDR is calculated as the ratio of number of input peaks over the number of IP peaks using sample swap [17]. To test whether the results depend on the peak caller used, we also used the findPeaks program from HOMER (http://homer.ucsd.edu/homer/ngs/peaks.html) to identify ER (via “-style factor” option) and H3K4me3 peaks (via “-style histone” option), with the cutoff of fold-change ≥ 2 over input and FDR ≤ 1E-4. FindPeaks showed good performance on the identification of histone modification peaks [27]. Peaks were filtered out if they overlap the blacklist (total 13.67 megabases), which is a collection of mappable regions with artificially high signal (https://sites.google.com/site/anshulkundaje/projects/blacklists). The blacklist was combined from a consensus list empirically defined by the Encyclopedia of DNA Elements (ENCODE) consortium, available at http://hgdownload.cse.ucsc.edu/goldenPath/hg19/encodeDCC/wgEncodeMapability/wgEncodeDacMapabilityConsensusExcludable.bed.gz, and the Terry’s blacklist primarily based on repeat annotations, available at http://mitra.stanford.edu/kundaje/akundaje/release/blacklists/hg19-human/Duke_Hg19SignalRepeatArtifactRegions.bed.gz.

### Motif finding

To identify ER and NRF1 DNA binding motifs, sequence spanning the peak center +/- 50 bp was extracted using the getfasta command in the Bedtools suite [28]. Motif was identified using the meme software (v4.8.1) [29], at the parameter settings described in [18].

### Duplicate level estimation and correlation between replicates

Number of raw reads in a peak was estimated from unique alignments both before and after duplicate removal, using the intersectBed command from the Bedtools suite. Number of duplicates represents the difference between the two estimates. Duplicate rate, defined as the ratio of the number of duplicates over the number of uniquely mapped reads, was estimated for peaks, non-peak regions and peak-flanking regions in IP, as well as for peak and non-peak corresponding regions in input. Non-peak regions represent the rest of the mappable genome that are not covered by peaks. To avoid the possible influence from peaks and consider the difficulty in defining the precise peak boundary, non-peak regions were 100 bp away from peaks, unless stated otherwise. Average duplicate rate in non-peak regions serves as the baseline in assessing duplicate rate in peaks. To understand whether peak adjacent regions are similarly enriched with duplicates as peaks or have a comparable duplicate rate as the average of non-peak regions, we also estimated duplicate rate for peak-flanking regions. They are peak 5’ and 3’ regions, and both were 300 bp away from the peak and had the same size as the peak. A 300-bp (~ the fragment size) separation was used to minimize influence from the peak. If the duplicate rate is much higher in the peaks compared with the baseline from non-peak regions and with that from peak-flanking regions, it is a strong indication that duplicates in peaks likely represent signal. In calculating the number of duplicates per base, the reads whose alignments start at the same position on the reference but on different strands were counted separately.

To calculate correlation between replicates in duplicate level, blacklist-filtered peaks from replicates were first merged into a single list if they show at least 1 bp overlap. For each merged peak, the number of duplicates was estimated (see above) in each replicate and normalized to per kb per 10 million uniquely mapped reads (RPK10M). Pearson correlation was computed using log_2_-transformed RPK10M values.

### Correlation between duplicate level and six features

For each IP, Spearman rank correlation was computed between duplicate level in peaks and each of the six features including non-duplicate level in peaks, duplicate and non-duplicate level in peak corresponding regions in input, peak GC content, as well as percentage of segmental duplication and low-complexity sequences in peaks. Duplicate level in peaks and in corresponding regions in input was estimated as the number of redundant reads per kb without library size normalization, and non-duplicate level was estimated similarly from nonredundant reads. GC content represents the number of guanine and cytosine bases divided by the total bases in a peak. Percentage of segmental duplication is the fraction of a peak that overlaps regions of segmental duplication, defined as those with > = 90% sequence identity over at least 1 kb (http://humanparalogy.gs.washington.edu/build37/build37.htm) [30]. Percentage of low-complexity sequence is the fraction of a peak that overlaps low complexity regions (https://figshare.com/articles/Low_complexity_regions_in_hs37d5/969685) [31].

### The prediction of natural duplicates in peaks

In predicting the proportion of duplicates as true signals in blacklist-filtered peaks, we recalculated its raw duplicate level (i.e., number of duplicates per kb) and performed the prediction based on target enrichment level (i.e. number of nonredundant reads per kb) using the "lowess()" function in R. To avoid overestimation, natural duplicate level was set at the raw or predicted level, whichever is smaller.

## Results and discussion

We first analyzed the three PCR-free H3K4me3 ChIP-seq data in HeLa. About two-fifths of the uniquely mapped reads were duplicates (S1 Table), and 97% of the duplicates were within the peaks that represented only 1.9% of the mappable genome. Of the other three datasets with PCR amplification from narrow-peak marks, the six NRF1 ChIP-seq data had 10.3–20.9 million uniquely mapped reads and 2.94–35.81% duplicates; the 13 ER ChIP-seq data had 18.5–79.3 million uniquely mapped reads, of which 6.76–24.10% were duplicates; and another 13 H3K4me3 ChIP-seq data had 27.1–50.1 million uniquely mapped reads including 20.19–55.38% duplicates (S1 Table). Focusing on the three datasets, we found that duplicates were highly enriched in peaks, especially within those having the highest confidence (lowest FDR), compared to the non-peak regions. Furthermore, duplicate level (duplicates per kb) in peaks was highly correlated between replicates and with the level of nonredundant reads. We conclude that a significant portion of duplicates in peaks represents true signal for narrow-peak marks. While H3K27me3 and H3K36me3 peaks were also enriched with duplicates, the duplicate level was substantially lower than that of narrow-peaks. For both marks, duplicates in peaks also largely represent signal.

### Genome-wide distribution of duplicates

**Fig 1:**
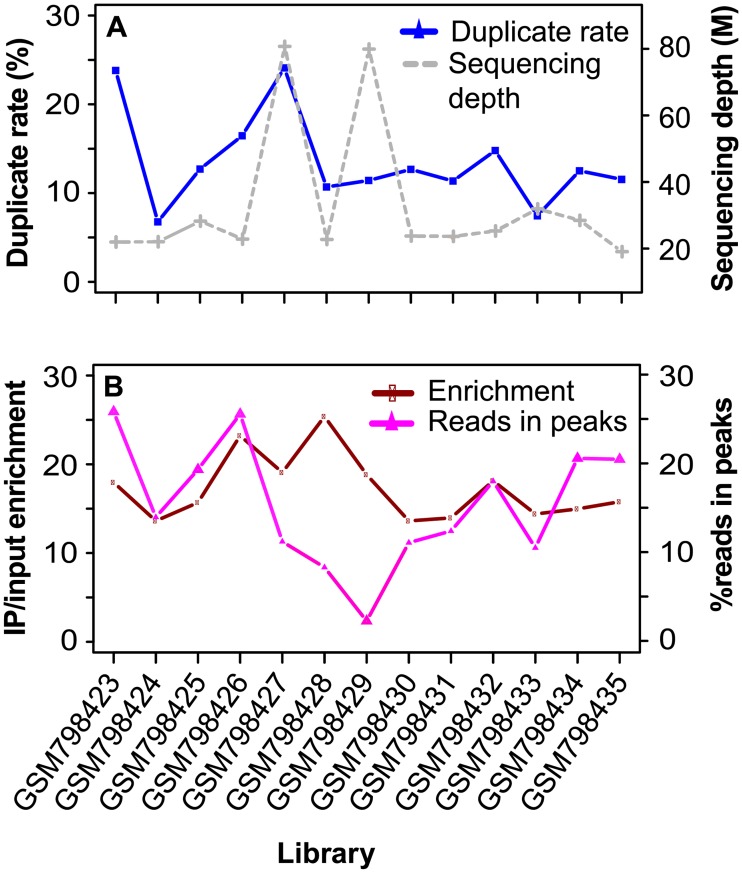
Duplicate rate versus sequencing depth and target enrichment level in ER ChIP-seq data. (**A**) Duplicate rate versus sequencing depth. (**B**) Enrichment level and the percentage of nonredundant reads in peaks. Duplicate rate was defined as the ratio of duplicate reads over uniquely mapped reads. Enrichment level was estimated as (number of nonredundant reads in peaks / total nonredundant reads in IP) / (number of nonredundant reads in peak-corresponding regions in input / total nonredundant reads in input).

For the ER dataset, 8.6% (median) of the positions with uniquely mapped reads had duplicates, versus only 2.0% (median) in the input libraries. GSM798423 and GSM798427, the two samples with the highest duplicate rates (24%) (Fig 1A), had at least twice as many positions with duplicates as the others. We further checked the per-base duplicates for those with > = 1 duplicate. Over 94% of the positions each had five or less duplicates, together contributing to approximately 79% of the total duplicates; about 1% had over ten duplicates (S1 Fig). As expected, the proportion of positions with duplicates was several times higher within narrow peaks than within broad peaks. Specifically, within ER, NRF1, and H3K4me3 peaks, 30.6, 45, and 40.3% of the positions with uniquely mapped reads had duplicates, versus only about 5% within H3K27me3 and H3K36me3 peaks.

We called peaks from uniquely mapped reads after duplicate removal and counted duplicates within peaks and non-peak regions (> = 100 bp away from peaks). We defined duplicate rate as the number of duplicates over total uniquely mapped reads, which is 3 to 11.5 times higher in ER peaks compared to the baseline in non-peak regions (S2 Fig). In contrast, duplicate rate was largely comparable (less than twofold difference) between their corresponding regions in input, with the exception of GSM798432 (2.3-fold) (S2 Fig). Representing about 0.5% of the mappable genome, ER peaks had a median of 14% of the nonredundant reads (Fig 1B) and 59.37% of the duplicates in IP (Table 1). The enrichment of duplicates in peaks is not due to the mapping artifact based on little overlap with the blacklist. On average, only 0.2% of the duplicates in peaks overlapped the blacklist, versus 8.9% in non-peak regions (Table 1).

**Table 1:** Duplicate level in ER peaks and non-peak regions.

Accession	A		B		C		D	
	Peak	Non-peak	Peak	Non-peak	Peak	Non-peak	Peak	Non-peak
GSM798423	60.98	39.02	0.19	3.46	0.05	0.44	0.78	0.16
GSM798424	71.25	28.75	0.45	28.24	0.06	0.44	0.39	0.02
GSM798425	71.79	28.21	0.04	11.50	0.03	0.41	0.57	0.05
GSM798426	80.68	19.32	0.05	22.27	0.04	0.45	0.66	0.04
GSM798427	53.26	46.74	0.02	3.60	0.02	0.33	1.62	0.17
GSM798428	47.63	52.37	0.01	15.47	0.03	0.49	0.72	0.06
GSM798429	26.89	73.11	0.03	9.62	0.06	0.39	1.61	0.09
GSM798430	39.70	60.30	0.02	16.57	0.04	0.43	0.55	0.08
GSM798431	59.37	40.63	0.02	19.56	0.05	0.45	0.66	0.05
GSM798432	55.12	44.88	1.91	4.89	0.34	0.42	0.55	0.09
GSM798433	48.98	51.02	0.05	12.84	0.06	0.39	0.40	0.04
GSM798434	73.99	26.01	0.03	11.23	0.03	0.40	0.55	0.04
GSM798435	67.71	32.29	0.38	8.41	0.07	0.43	0.46	0.05

A, percentage of total library duplicates in peaks and non-peak regions. B, percentage of duplicates in peaks and non-peak regions that overlaps the blacklist. C, percentage of positions with at least one duplicate that overlaps the blacklist. D, average number of duplicates per position, calculated as the ratio of total duplicates over the total number of positions with at least one uniquely mapped read, with positions overlapping the blacklist excluded. Only uniquely mapped reads with a minimum mapping quality score of 20 and no mismatch at the first five bases were used. Non-peak regions, the bases not covered by peaks.

Similar to the ER data, the H3K4me3 and NRF1 samples also had a high FRiP (fraction of reads in peaks), which is a ChIP-seq quality metric of global enrichment proposed by the ENCODE Consortium [5], and high proportion of duplicates in peaks (Table 2). For example, H3K4me3 peaks represented 2% of the mappable genome but had over 65% of the nonredundant reads (Table 2). Accordingly, H3K4me3 peaks contained over 82% of the total duplicates, showing a 15-fold median enrichment over the corresponding regions in inputs (Table 2).

**Table 2:** Enrichment of duplicates in H3K4me3 and NRF1 peaks.

Accession	Duplicate rate (%)	Peak size (%)	Nonredundant reads			Duplicates		
			IP (%)	Input (%)	Ratio	IP (%)	Input (%)	Ratio
GSM1233880	20.19	2.15	64.60	2.80	23.07	95.67	2.48	38.58
GSM1233881	26.19	2.03	77.33	2.57	30.09	96.73	2.25	42.99
GSM1233905	53.96	1.97	75.25	7.11	10.58	82.11	6.51	12.61
GSM1233906	32.75	1.99	82.52	7.26	11.37	93.67	6.62	14.15
GSM1233907	37.78	1.99	81.96	7.36	11.14	91.23	6.70	13.62
GSM1233926	21.08	1.79	69.07	5.32	12.98	94.02	4.28	21.97
GSM1233927	29.32	1.73	80.65	5.12	15.75	97.90	4.11	23.82
GSM1233947	55.38	1.95	77.28	6.34	12.19	83.71	5.81	14.41
GSM1233948	30.70	1.94	83.01	6.23	13.32	94.20	5.68	16.58
GSM1233949	34.94	2.10	81.81	6.86	11.93	89.50	6.28	14.25
GSM1233969	53.30	1.91	80.23	6.11	13.13	86.73	5.78	15.01
GSM1233970	29.32	1.95	82.95	6.10	13.60	93.62	5.75	16.28
GSM1233971	40.80	2.17	81.16	6.73	12.06	88.49	6.36	13.91
GSM2574769	23.57	0.28	25.1	0.98	25.61	81.38	2.16	37.68
GSM2574770	8.77	0.11	2.89	0.45	6.42	30.06	1.1	27.33
GSM2574771	21.21	0.24	21.4	0.88	24.32	79.49	1.96	40.56
GSM2574780	35.81	0.15	26.96	0.49	55.02	48.34	0.75	64.45
GSM2574812	17.86	0.16	19.91	0.51	39.04	91.61	0.97	94.44
GSM2574813	2.94	0.06	0.88	0.22	4	29.08	0.43	67.63

Peaks were called by MACS using uniquely mapped reads after duplicate removal, and those overlapping the blacklist were filtered out. Only reads with a minimum mapping quality score of 20 and no mismatch at the first five bases were included in the analysis. Duplicate rate (%), number of duplicates divided by the number of uniquely mapped reads. Peak size (%), total peak size over the size of mappable genome (0.75 x genome size). Nonredundant reads in IP (%), number of nonredundant reads in peaks over total nonredundant reads in IP (FRiP). Nonredundant reads in input (%), number of nonredundant reads in peak-corresponding regions in input over total nonredundant reads in input. Proportion of duplicates in peaks and in peak-corresponding regions from input was calculated similarly. H3K4me3, the first 13 samples from GSM1233880 to GSM1233971; NRF1, the bottom six samples from GSM2574769 to GSM2574813. See S1 Table for sample information.

Next, we used the ER dataset to examine how the top positions with the most duplicates distribute in the genome. We extracted the top 2,000, 5,000 and 10,000 positions and analyzed their genomic locations. Between 35.7 and 97.93% (median 84.7%) of the positions were located in the ER peaks (S3 Fig), with the lowest (35.7 and 53.42%) from GSM798429 and GSM798430, the two samples that had the highest fractions (>60%) of duplicates in non-peak regions (Table 1). We thus predict that positions within peaks should have more duplicates than those in non-peak regions. To confirm it, we estimated the average number of duplicates per base, which was 4.9–19.5 times higher in peaks than in non-peak regions (Table 1). In parallel, we extracted the top 10,000 positions from both peak and non-peak regions and compared the number of duplicates per base. Except GSM798429 and GSM798430 described above, the other 11 samples showed 1.5- to 5.8-fold enrichment in peaks (S4 Fig).

**Fig 2:**
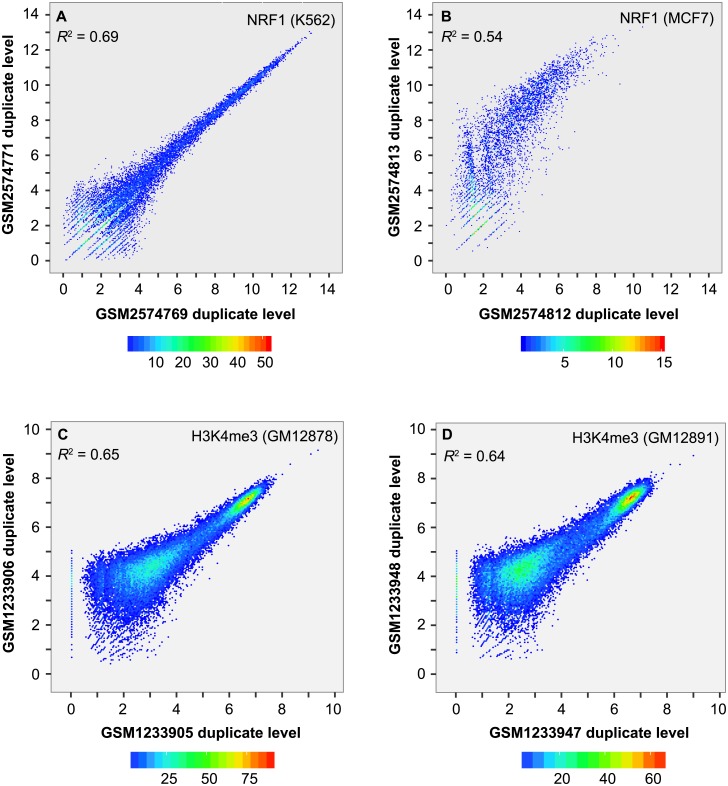
Scatter plot of duplicate level within peaks between replicates. (**A**) NRF1 in K562. (**B**) NRF1 in MCF7. (**C**) H3K4me3 in GM12878. (**D**) H3K4me3 in GM12891. Peaks overlapping the blacklist were filtered out. Duplicate level was estimated as the number of duplicates per kb per 10 million uniquely mapped reads and log_2_ transformed. *R*^2^ value was calculated using Pearson correlation.

Finally, we checked whether duplicate level within peaks is correlated between replicates. We merged peaks from replicates, estimated the number of duplicates per merged peak and normalized to reads per kb per 10M (RPK10M). Pearson correlation, calculated using RPK10M on log_2_ scale, varied between 0.722 (95% confidence interval (CI): 0.719–0.726) and 0.832 (95% CI: 0.829–0.834) for ER (S5 Fig), between 0.732 (95% CI: 0.722–0.741) and 0.833 (95% CI: 0.828–0.837) for NRF1 (Fig 2A and 2B), and between 0.740 (95% CI: 0.736–0.745) and 0.874 (95% CI: 0.871–0.876) for H3K4me3 (Fig 2C and 2D). The noticeable enrichment of duplicates in peaks and high correlation of duplicate level between replicates suggested that duplicates in ER, NRF1 and H3K4me3 peaks largely represent true signals rather than artifacts.

### Duplicates are over enriched in highly confident peaks

We have found that duplicates are enriched in peaks relative to the non-peak regions. Next we ask whether the most confident peaks are more enriched with duplicates, particularly for narrow-peak marks, and whether peak-flanking regions are also enriched with duplicates. To answer both questions, we split peaks into 10 equal-sized groups according to the FDR, with peaks in group 1 having the lowest FDR (the highest confidence) and those in group 10 having the highest FDR.

**Fig 3:**
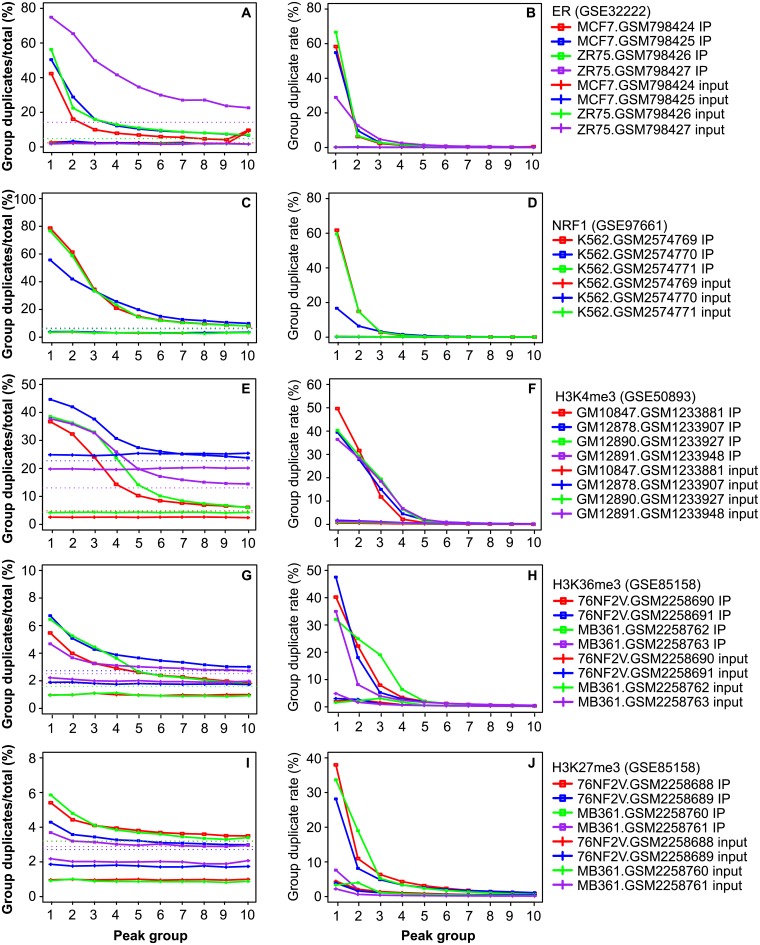
Plot of duplicate abundance versus peak confidence. (**A,C,E,G,I**) Duplicate rate in 10 groups of peaks and in the corresponding regions in input. (**B,D,F,H,J**) Proportion of total library duplicates in each of the groups and in the corresponding regions in input. Peaks were sorted based on *p* value in ascending order and split into 10 equal-sized groups, with group 1 having the smallest *p* values. *Y*-axis in the left panels represents duplicate rate per group, i.e., the number of duplicates over the total uniquely mapped reads in a group, as defined in Fig 1 legend. *Y*-axis in the right panels represents the proportion of total duplicates from a library in each group and in the peak-corresponding regions in input. The dotted horizontal lines in the left panels denote duplicate rates in the non-peak regions in IP (> = 100 bp away from peaks). See S1 Table for sample information.

For narrow-peak marks, group 1 had a duplicate rate of about 50% (31.09–78.86%), which decreased from group 2 to 10 (Fig 3A, 3C and 3E). However, in the corresponding regions from the input, all groups had similarly lower duplicate rates (Fig 3A, 3C and 3E). A similar pattern was observed for the proportion of duplicates across the 10 groups (Fig 3B, 3D and 3F). For example, group 1 peaks from ER had 11.59–66.61% of the total library duplicates, representing 43.11–82.91% of the duplicates from all peaks. In contrast, group 5 to 10 each had less than 1.5% of total library duplicates, similar to the corresponding regions in input libraries. This analysis revealed that, for narrow-peak marks, duplicates are much more abundant in the peaks with the highest confidence.

On the other hand, for H3K27me3 and H3K36me3, group 1 represents 0.2–2% of the mappable genome. In over 80% of the cases, it had >20% of the duplicates in a sample (Fig 3H, 3J; S6B, S6D, S6F, S7B, S7D and S7F Figs), suggesting that broad peaks are also enriched with duplicates. Nevertheless, due to the overall low duplicate rate, group 1 often had only a few percent of duplicates, which, in many of the cases, was not markedly higher than that of the other groups (Fig 3G, 3I; S6A, S6C, S6E, S7A, S7C and S7E Figs).

As narrow peaks were highly enriched with duplicates, we further examined the distribution of top 10,000 positions from ER peaks. They were from 196–408 peaks (S2 Table), with over 85% from only 100 peaks, indicating that the top positions tend to cluster together. Overall, those peaks had high confidence (small FDR) (S8 Fig) and often contained the ER binding motif. About 72–87% of these peaks contained the ER binding motif, showing more than twofold enrichment over randomly selected peaks. Similar patterns were observed for the top 2,000 and 5,000 positions (S2 Table).

Based on the above analysis, we finally checked the flanking regions from ER peaks in group 1 (with the highest duplicate rate) to see whether they are also enriched for duplicates, using those from group 5 (generally lack enrichment of duplicates) as the baseline. We defined the flanking regions as peak 5’ and 3’ regions that are 300 bp away from the peak and have the same size as the peak. We further split group 1 and 5 into 10 equal-sized subgroups as described above. Regardless of the duplicate rates in peaks, the flanking regions had similar duplicate rates across all the subgroups, which were markedly lower than those of the peaks (S9A–S9D Fig). Thus, duplicates appear to be exclusively enriched in peaks.

### Impact of duplicates on peak calling and signal quantification

As duplicate rate was on average over seven times higher in narrow peaks than in broad peaks, we assessed the extent by which duplicate removal impacts peak calling in ER, H3K4me3 and NRF1. Unique alignments were filtered by requiring a mapping quality score of at least 20 and no mismatch over the first five bases at the 5’ end. We called peaks with and without duplicate removed and estimated the portion of peaks unique to either of the two options. We found that the proportion of library duplicates in ER peaks (merged from the two options and filtered by blacklist) was positively correlated with the proportion of peaks unique to duplicate removal (Spearman rank correlation *R* = 0.66, *p* = 1.71e-02) and negatively correlated with the proportion of peaks unique to no duplicate removal (*R* = -0.80, *p* = 1.84e-03). The correlation was more obvious for H3K4me3 (*R* = 0.96, *p* = 2.20e-16; *R* = -0.98, *p* = 2.20e-16).

There were 5% ER peaks unique to duplicate removal and 2.72% unique to no duplicate removal (S3 Table). To understand whether these unique peaks represent true binding sites or false positives, we used the meme program to scan the 100-bp sequence spanning the peak center for matches to the ER binding motif. On average, 22.5% and 24.5% of the two unique peak sets contained the ER motif (S3 Table), indicating that at least a subset represents true binding sites. For NRF1 in HepG2, K562 and MCF7, there were 5.32% and 4.05% peaks unique to duplicate removal and no duplicate removal, respectively (S4 Table). As over 60% of the NRF1 binding sites are located within the -150 to 50 bp regions around transcription start sites (TSSs) [32], we checked overlap with the TSS ± 2kb regions in the Ensembl v78 annotation and with the H3K4me3 peaks from reference epigenome in HepG2, K562 and HMEC [33]. Of the unique peaks from duplicate removal, 24.5% had the NRF1 binding motif, and 60.5 and 70.9% overlapped the TSS ± 2kb regions and H3K4me3 peaks, versus 33.3, 33.5 and 37.3% of the unique peaks from no duplicate removal (S4 Table).

Similarly, there were 6.23% H3K4me3 peaks unique to duplicate removal and 1.43% unique to no duplicate removal (S5 Table). H3K4me3 is a hallmark of promoters. To assess what proportion of those unique peaks might represent true H3K4me3 sites, we intersected both shared (called with both options) and unique sites with the 4-kb windows centered on the TSSs. Of the H3K4me3 sites unique to either option, about 22% showed overlap (S5 Table), versus about 51% for the shared peaks. Intersecting with H3K4me3 peaks from the GM12878 reference epigenome [33] revealed 54% overlap for unique peaks and 85% overlap for shared peaks. Complete removal of duplicates is currently a common practice for ChIP-seq. It had 386–1,385 unique peaks but missed 2,123–5,793 peaks that are unique to the option of no duplicate removal in five of the samples (S5 Table). These samples generally had high duplicate rates than the others. About half of the peaks unique to no duplicate removal overlapped with H3K4me3 peaks from the GM12878 reference epigenome. Thus, complete deduplication is not an ideal option, in particular for the five samples. On the other hand, as some of the duplicates represent PCR artifacts, it is necessary to develop a method to remove noise duplicates, which is expected to minimize false positives in peak detection.

To investigate whether the results depends on the peak caller used, we also identified ER and H3K4me3 peaks using findPeaks from HOMER. In terms of the proportion of peaks unique to duplicate removal and to no duplicate removal, the pattern was highly consistent between MACS and findPeaks (S3 and S5 Tables). For example, GSM798428 and GSM798430 had comparable number of unique ER peaks before and after duplicate removal for both callers, while GSM798427 had over three times more peaks unique to duplicate removal (S3 Table). Apparently, analysis of the three narrow-peak marks indicates that the option of duplicate removal impacts peak calling and the extent of impact tends to be library dependent. Thus, it is advisable to develop an optimal deduplication strategy to achieve both high sensitivity and specificity in peak detection.

Finally, ER data was used to understand the impact of duplicate removal on signal quantification. For peaks in group 1 and 5 (see above), we calculated their RPK10M for IP and input and plotted the input-subtracted RPK10M on log_2_ scale. We observed 1.21- to 2.47-fold changes before and after duplicate removal for peaks in group 1 (S10 Fig), but no obvious differences for peaks in group 5. As expected, duplicate removal mainly reduces the signals in highly confident peaks.

### Factors associated with duplicate level

To separate duplicates within peaks into those that likely represent true signal and others that are PCR amplification noise, we need to understand what factors are associated with the duplicate level. We first considered sequencing depth. For the same IP, sequencing at a higher depth likely increases the chance of generating duplicates. However, confronted by factors such as difference in the amount of starting material and immunoprecipitation efficiency, sequencing depth does not necessarily correlate well with duplicate rate across different libraries. For example, GSM798427 and GSM798429 from the ER dataset had the highest sequencing depths (79.3 and 78.3 million), their duplicate rates differed by twofold (24.10% versus 11.42%) (Fig 1A). This is likely because GSM798429 had only 6,600 peaks (S3 Table). GSM798423, on the other hand, had only 21.4 million reads but a high duplicate rate (23.81%) (Fig 1A).

**Fig 4:**
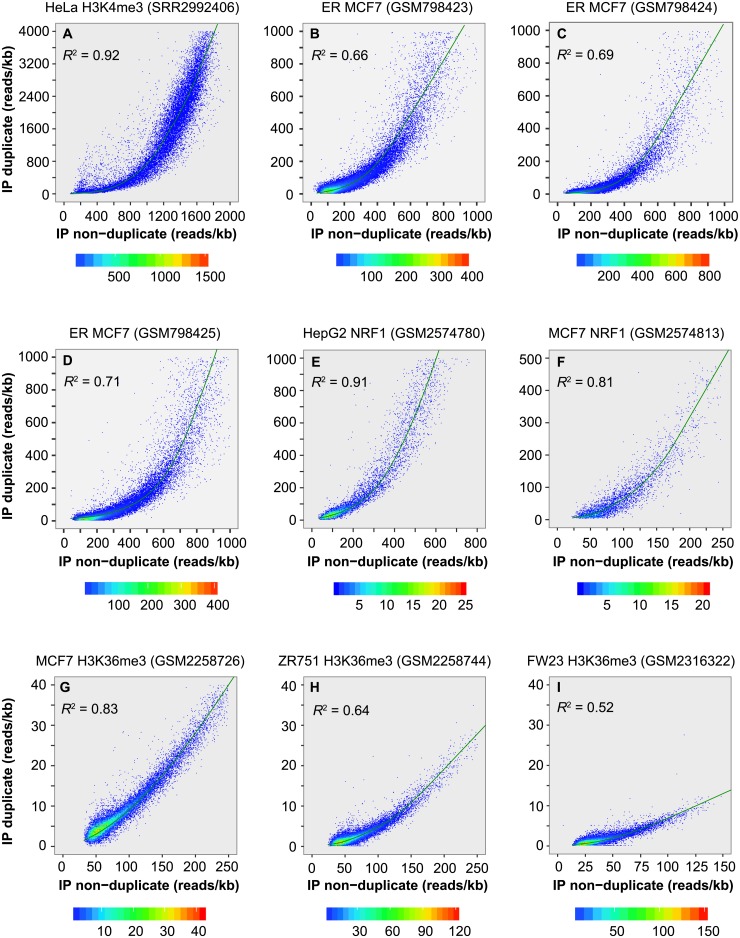
Duplicate level in peak is correlated with mark enrichment. (**A**) PCR-free H3K4me3 ChIP-seq data in HeLa cell line. (**B**-**D**) ER ChIP-seq data in MCF7 cell line. (**E**,**F**) NRF1 ChIP-seq data in HepG2 and MCF7 cell line. (**G**-**I**) H3K36me3 ChIP-seq data in MCF7 and ZR751 cell line and in fetal retinal tissue. Peaks were called from alignments with duplicate removed. *X*-axis indicates mark enrichment level in peaks, estimated as the number of nonredundant reads per kb, and y-axis shows the number of duplicates per kb. The curve was constructed using the "lowess()" function in R. *R*^2^ value was calculated using Spearman rank correlation.

**Fig 5:**
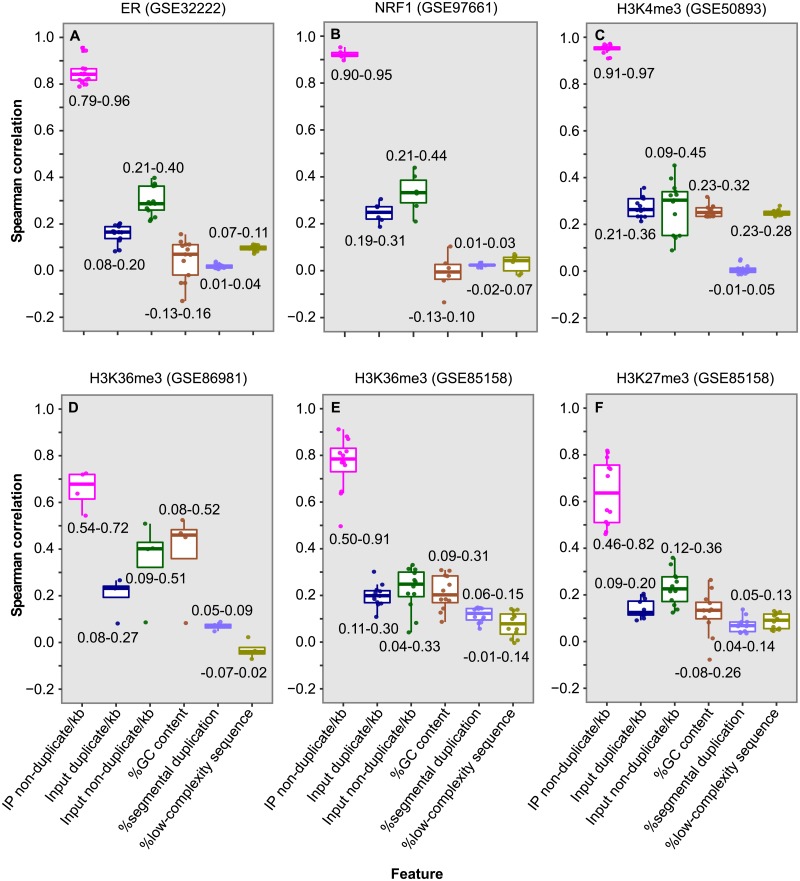
Box plot of Spearman rank correlation between duplicate level in peak and six factors. (**A**) Thirteen ER libraries in breast cancer cell lines. (**B**) Six NRF1 libraries, including one in HepG2, two in MCF7 and three in K562. (**C**) Thirteen H3K4me3 libraries in lymphoblastoid cell lines. (**D**) Four H3K36me3 libraries in fetal retinal tissue. (**E**) Twelve H3K36me3 libraries in breast cancer cell lines. (**F**) Twelve H3K27me3 libraries in breast cancer cell lines. For each peak, duplicate level was estimated as the number of duplicates divided by peak size in kb, and non-duplicate level was estimated similarly. Duplicate and non-duplicate levels in peak corresponding regions in input were also calculated. GC content represents the number of guanine and cytosine bases divided by the total bases in a peak. Percentage of segmental duplication is the proportion of a peak that overlaps regions of segmental duplication, defined as those with > = 90% sequence identity over at least 1 kb (http://humanparalogy.gs.washington.edu/build37/build37.htm) [30]. Percentage of low-complexity sequence is the proportion of a peak that overlaps low complexity regions (https://figshare.com/articles/Low_complexity_regions_in_hs37d5/969685) [31].

We next examined whether duplicate level is correlated with non-duplicate level in peaks. Uniquely mapped reads in each peak were split into duplicates, which are redundant reads mapped to the same location and strand, and non-duplicates (nonredundant reads). Duplicate level and non-duplicate level were estimated as the number of redundant and nonredundant reads per kb, respectively. We found that, within peaks, duplicate level was highly correlated with non-duplicate level for ER (Spearman rank *R* = 0.79–0.96) (Figs 4B–4D and 5A), NRF1 (*R* = 0.90–0.95) (Figs 4E, 4F and 5B), PCR-free H3K4me3 in HeLa (*R* = 0.96) (Fig 4A) and H3K4me3 in lymphoblastoid cell lines (*R* = 0.91–0.97) (Fig 5C and S11A and S11B Fig). Obvious correlation was also detected for the two broad-peak marks, H3K27me3 (*R* = 0.46–0.86) (Fig 5F and S11C–S11F Fig) and H3K36me3 (*R* = 0.50–0.91) (Figs 4G–4I and 5D, 5E). As expected, these two variables were much less correlated for the peak corresponding regions in inputs, as showed for the ER dataset (*R* = 0.12–0.33) (S12 Fig). The high correlation indicates that we can predict the duplicate level in peaks as belonging to signal based on the non-duplicate level, which will allow us to properly allocate duplicates between signal and noise.

To understand whether additional factors are also associated with duplicate level, we also assessed the correlation between duplicate level in peaks and five other variables (Fig 5). The analysis revealed a modest correlation with non-duplicate level in the peak corresponding regions in input (*R* = 0.04–0.51; median 0.28), likely reflecting the influence of local chromatin structure. In general, a smaller correlation (median *R* = 0.11) was detected with the other four variables including duplicate level in peak corresponding regions in input, %GC content, %segmental duplication and %low-complexity sequences in peaks. Therefore, of the six factors examined, non-duplicate level is the most critical determinant of duplicate level in peaks.

### Partition of duplicates in peaks

To separate duplicates in peaks into true signal and noise, we first examined the duplicate frequency at the positions that had at least one duplicate in peaks. Ninety-six to ninety-nine point eight percent of the positions in H3K4me3 peaks and 86.5–96.4% of the positions in ER peaks had 1–5 duplicates. To minimize noise, we arbitrarily kept a maximum of five duplicates per position. We then recalculated the duplicate level (number of duplicate per kb) for each peak and obtained the predicted level based on the peak enrichment level (number of nonredundant reads per kb) (Fig 4A–4I and S11A–S11F Fig).

**Fig 6:**
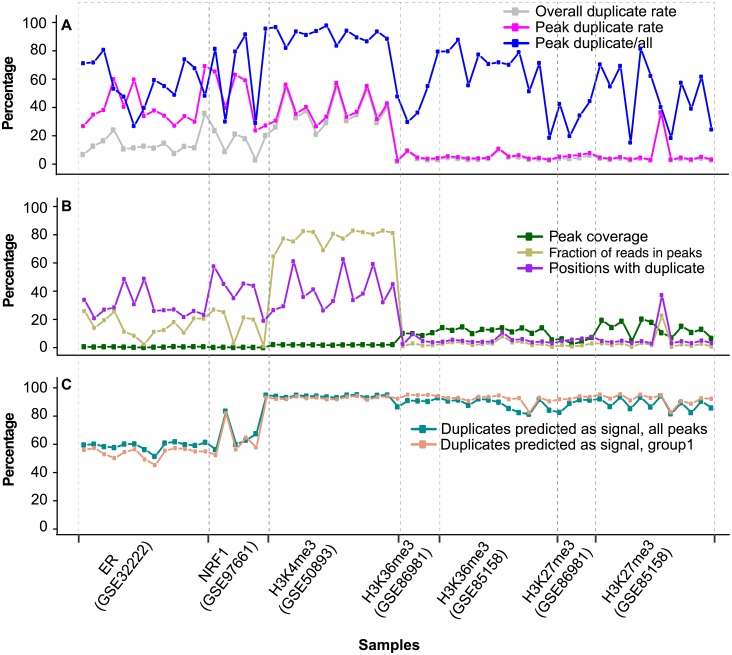
Prediction of duplicates as signal based on peak enrichment. (**A**) Duplicate rate in a library and in peaks and proportion of duplicates in peaks. Duplicate rate in a lib was estimated as the number of duplicates divided by the number of uniquely mapped reads. Duplicate rate in peaks was estimated in the same way. (**B**) Plot of peak coverage, fraction of positions with duplicates and fraction of nonredundant reads in peaks. Peak coverage was estimated as the total peak size over the mappable genome size (0.75 x genome size). Fraction of positions with duplicates was estimated as the number of positions with duplicates over the number of positions with uniquely mapped reads. Fraction of reads in peaks (FRiP), fraction of uniquely-mapped, nonredundant reads in peaks. (**C**) Proportion of duplicates predicted as signal. The prediction was based on the correlation between peak duplicate and non-duplicate level, as showed in Fig 4.

Overall, 51.3–61.7% of the duplicates in ER, 61.3–94% in NRF1 and 92.7–95% in H3K4me3 peaks should represent true signal (Fig 6C). For the top 10% of the peaks, a slightly lower proportion was predicted to be true signal (Fig 6C). The proportion of duplicates predicted as signal (Fig 6C) is well correlated with the enrichment level (the fraction of nonredundant reads in peaks, FRiP) (Fig 6B), the proportion of positions with duplicates (Fig 6B), and the proportion of duplicates within peaks (Fig 6A). Compared to the above narrow-peak marks, H3K27me3 and H3K36me3 peaks had over 10 times larger coverage (Fig 6B) and over 10 times less enrichment (FRiP) (Fig 6B). Consequently, within peaks the duplicate rate (Fig 6A) and the proportion of mapped positions with duplicates (Fig 6B) were only ~5%. Nevertheless, about half of the duplicates were located within peaks (Fig 6A), and over 80% of them were predicted to be true signal (Fig 6C).

**Fig 7:**
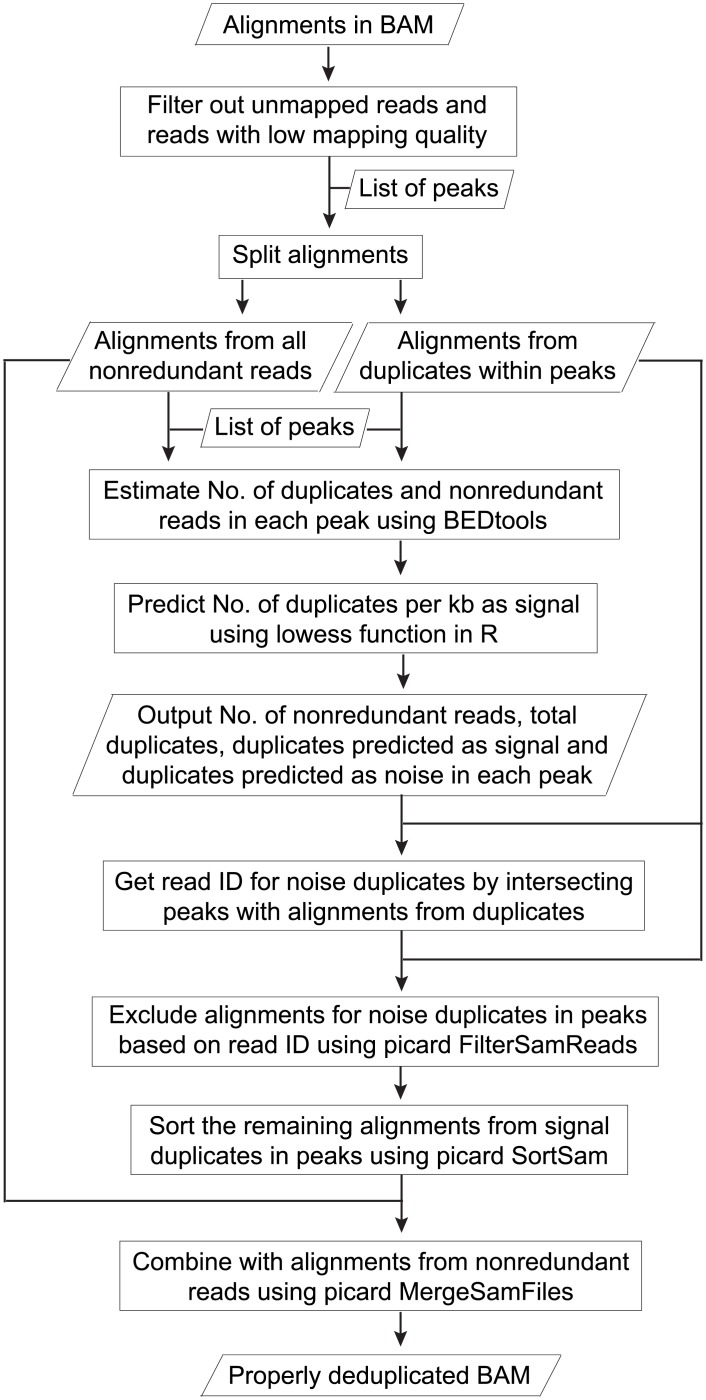
Flowchart for optimal deduplication in peaks. The workflow takes a BAM file and a list of peaks as input. It outputs a table that shows the number of nonredundant reads (non-duplicates), duplicates predicted as signal and duplicates as noise for each peak. A properly deduplicated BAM file is also generated, which contains alignments for all nonredundant reads and for duplicates in peaks that are predicted as signal. For each peak, if *N* represents the predicted number of noise duplicates and *S* represents the predicted number of signal duplicates, a list of *N* read ID is randomly extracted from *N*+*S* duplicates mapped to that peak. Alignments for the noise duplicates are then excluded, and alignments for the remaining duplicates are combined with those from nonredundant reads.

We proposed a strategy for optimal deduplication in peaks (Fig 7). Based on the high correlation between duplicate level and the level of nonredundant reads in peaks, the number of duplicates as signal could be predicted using local regression. On the other hand, for the non-peak regions (regions not covered by peaks) and input, the current practice of complete duplicate removal can be applied. As peaks from narrow-peak marks had a much higher duplicate rate (Fig 6A) and FRiP (Fig 6B) than those from broad-peak marks, optimal deduplication would be much less beneficial for the latter. We have provided scripts for the automation of complete duplicate removal in non-peak regions and proper deduplication in peaks. The properly deduplicated BAM file and the list of peaks with number of nonredundant reads, signal and noise duplicates could be easily used for downstream analysis. The scripts are available at GitHub (https://github.com/shulantianmayo/dedup).

## Conclusion

ChIP-seq data contains redundant reads, which are a mixture of PCR artifacts and natural duplicates. Currently, duplicates are filtered out prior to peak calling and signal quantification. Using public ER, NRF1 and H3K4me3 ChIP-seq data, we demonstrate that the current practice of filtering out all duplicates in peaks introduces a strong bias for narrow-peak marks, leading to a preferential loss of signals in highly confident peaks. However, the bias is less obvious for H3K27me3 and H3K36me3.

For the three narrow-peak datasets, duplicates are predominantly located in peaks, especially within those with high confidence, but at the baseline level in the flanking regions. The same pattern of duplicate enrichment was not observed in the corresponding regions in the inputs. In addition, duplicate level (number of duplicates per kb) in peaks is well correlated between replicates and with the enrichment level (nonredundant reads per kb). Collectively, these evidence supports that a substantial portion of the duplicates in peaks represents true signals.

Our analysis argues for the development of a more appropriate approach to handle duplicates in peaks, especially for narrow-peak marks, rather than simply filtering out all duplicates. The strategy is based on the high correlation of the duplicate level in peaks with the level of target enrichment. As demonstrated in this study, a substantial portion of duplicates in peaks is predicted to represent true signals that should be retained for downstream analysis.

## Supporting information

S1 Fig Proportion of positions with different number of duplicates in ER ChIP and input libraries. GSM798423 to GSM798435 are IP and the other five are inputs. Only positions with at least one duplicate were included in the analysis. Ninety-four point six percent (median) of the positions had no more than three duplicates.
(PDF)

S2 Fig Duplicate rate in ER peaks and non-peak regions and in the corresponding regions in inputs. Non-peak regions are the rest of the mappable genome that are 100 bp away from peaks.
(PDF)

S3 Fig A large proportion of top positions in the ER libraries are from peaks. For each library, the top 2,000, 5,000 and 20,000 positions with the highest number of duplicates were analyzed. The overall duplicate rate was also plotted for each library.
(PDF)

S4 Fig Box plot of the number of duplicates per position. The top 10,000 positions with the most duplicates from both ER peaks and non-peak regions were analyzed. Non-peak regions are those not covered by peaks.
(PDF)

S5 Fig Scatter plot of duplicate level within ER peaks between replicates. Duplicate level was estimated as the number of reads per 10 million (RPK10M) on log2 scale. Breast cancer cell lines BT-474 (left) and TAM-R (right) were shown. See Fig 2 legend for details.
(PDF)

S6 Fig Duplicate rate versus confidence level of H3K36me3 peaks. (**A,C,E**) Duplicate rate in 10 groups of peaks and in the corresponding regions in input. The dotted horizontal lines denote duplicate rates in the non-peak regions (> = 100 bp away from peaks). (**B,D,F**) Proportion of total library duplicates in each of the groups and in the corresponding regions in input. See Fig 3 legend for details.
(PDF)

S7 Fig Duplicate rate versus confidence level of H3K27me3 peaks. (**A,C,E**) Duplicate rate in 10 groups of peaks and in the corresponding regions in input. The dotted horizontal lines denote duplicate rates in the non-peak regions (> = 100 bp away from peaks). (**B,D,F**) Proportion of total library duplicates in each of the groups and in the corresponding regions in input. See Fig 3 legend for details.
(PDF)

S8 Fig Positions with the most duplicates tend to present in highly confident ER peaks. Peaks were ranked (1 to 100) based on *p* value, with rank 1 indicating the top 1% of the peaks with the smallest *p* values. For each library, the top 10,000 positions with the most duplicates were identified from peaks, and the ranks of the peaks covering these positions were plotted. The top 10,000 positions were from relatively lower confident peaks in GSM798427 and GSM798429; they had over 2.5-fold more uniquely mapped reads than the other 11 libraries.
(PDF)

S9 Fig Duplicate rate in ER peaks and the flanking regions. Peaks from group 1 (top 10% peaks with the lowest *p* values) and 5 (the fifth decile) were both split into 10 subgroups of equal size. Non-peak regions are 100 bp away from peaks. Flanking regions are peak 5’ and 3’ regions that are 300-bp away from the peaks and have the same size as the peaks. See S1 Table for sample information.
(PDF)

S10 Fig Box plot of the number of reads in ER peaks with and without duplicate removal. Peaks were called with duplicate removal and the top 10% peaks with the smallest *p* values were shown. Number of raw reads in peaks was estimated from alignments both before and after duplicate removal. *Y*-axis indicates the input-subtracted number of reads per 10 million (RPK10M) on log2 scale. Only reads with a minimum mapping quality score of 20 and no mismatch at the first five bases were used.
(PDF)

S11 Fig Scatter plot of duplicate versus non-duplicate level within H3K4me3 and H3K27me3 peaks. (**A**,**B**) H3K4me3 in lymphoblastoid cell lines. (**C**,**D**) H3K27me3 in fetal retinal tissue. (**E**,**F**) H3K27me3 in breast cancer cell lines. The curve was generated using the "lowess()" function in R. The coefficient of determination (*R*^2^) was calculated using Spearman rank coefficient. See Fig 4 legend for details.
(PDF)

S12 Fig Duplicate level versus non-duplicate level in ER peak corresponding regions in input. The coefficient of determination (*R*^2^) was calculated using Spearman rank coefficient. See Fig 4 legend for details.
(PDF)

S1 Table Public ChIP-seq data used in this study. (PDF)

S2 Table Number of ER peaks with the most highly duplicated positions. (PDF)

S3 Table Number of ER peaks called with and without duplicate removal. (PDF)

S4 Table Number of NRF1 peaks called with and without duplicate removal. (PDF)

S5 Table Number of H3K4me3 peaks called with and without duplicate removal. (PDF)
