# Supplementary material for: Identification of factors associated with duplicate rate in ChIP-seq data
Source: PLoS One. 2019 Apr 3;14(4):e0214723. doi: 10.1371/journal.pone.0214723 (PMC6447195; doi:10.1371/journal.pone.0214723)
Supplement: S1 Fig — GSM798423 to GSM798435 are IP and the other five are inputs. Only positions with at least one duplicate were included in the analysis. Ninety-four point six percent (median) of the positions had no more than three duplicates. (PDF) [file pone.0214723.s001.pdf]

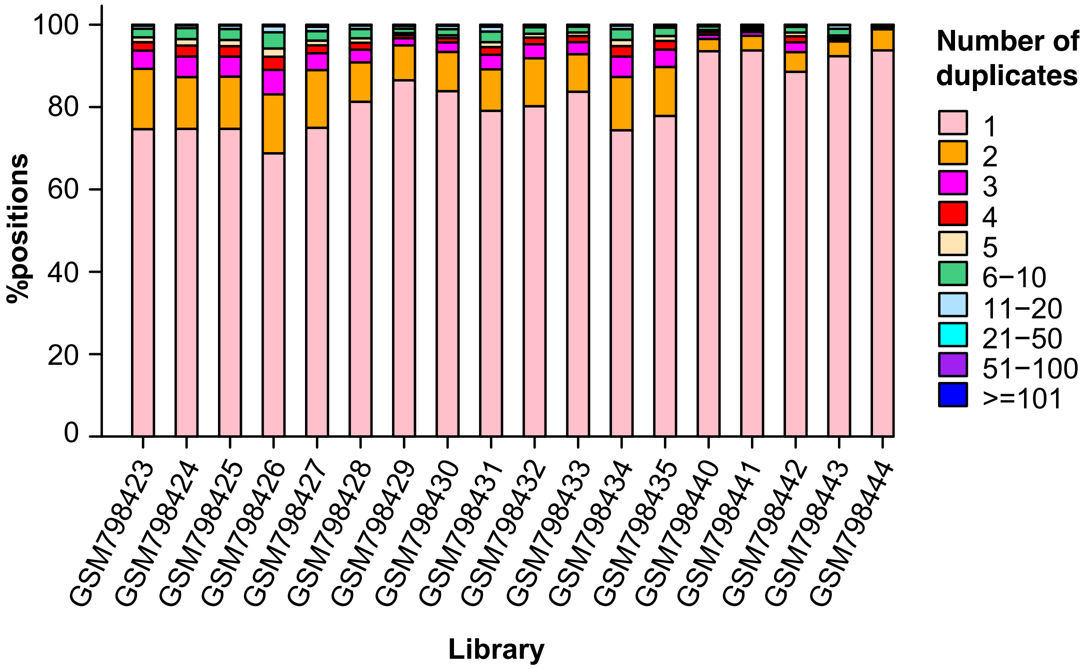

S1 Fig. Proportion of positions with different number of duplicates in ER ChIP and input libraries. GSM798423 to GSM798435 are IP and the other five are inputs. Only positions with at least one duplicate were included in the analysis. Ninety-four point six percent (median) of the positions had no more than three duplicates.
