# Supplementary material for: Identification of factors associated with duplicate rate in ChIP-seq data
Source: PLoS One. 2019 Apr 3;14(4):e0214723. doi: 10.1371/journal.pone.0214723 (PMC6447195; doi:10.1371/journal.pone.0214723)
Supplement: S2 Fig — Non-peak regions are the rest of the mappable genome that are 100 bp away from peaks. (PDF) [file pone.0214723.s002.pdf]

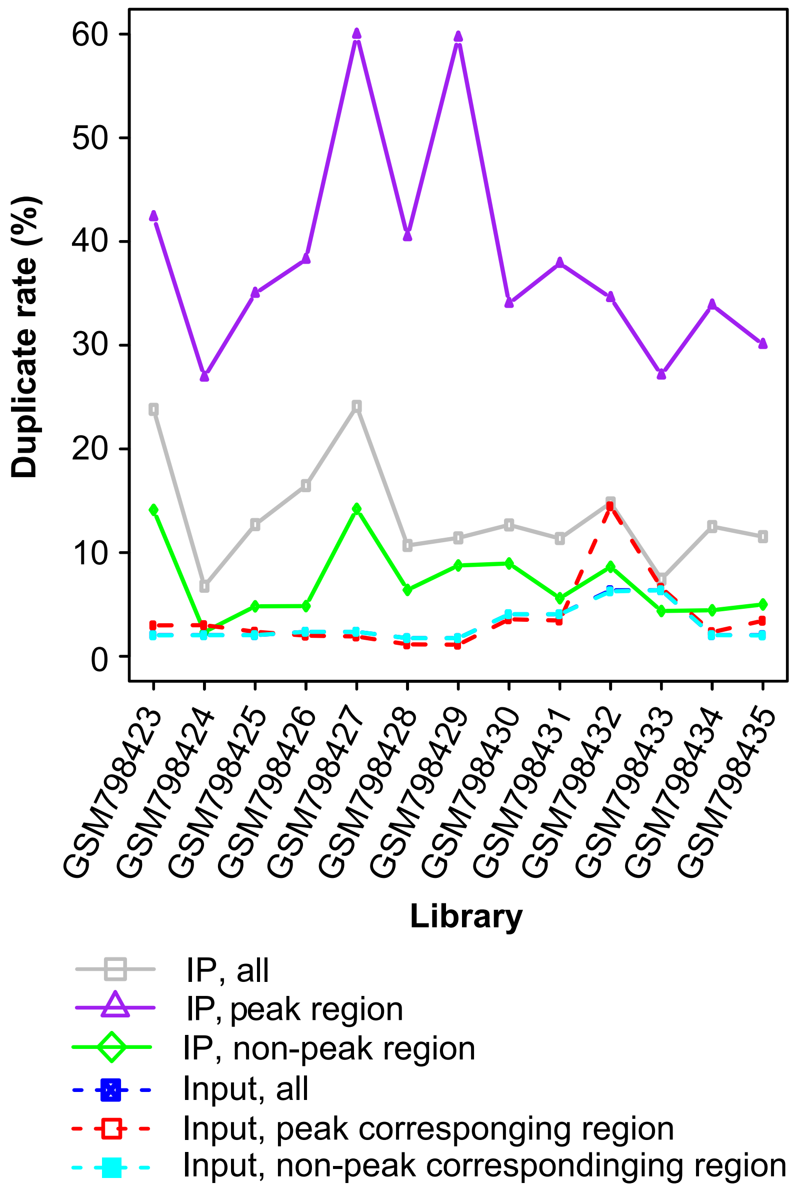

S2 Fig. Duplicate rate in ER peaks and non-peak regions and in the corresponding regions in inputs. Non-peak regions are 100 bp away from peaks.
