# Supplementary material for: Identification of factors associated with duplicate rate in ChIP-seq data
Source: PLoS One. 2019 Apr 3;14(4):e0214723. doi: 10.1371/journal.pone.0214723 (PMC6447195; doi:10.1371/journal.pone.0214723)
Supplement: S3 Fig — For each library, the top 2,000, 5,000 and 20,000 positions with the highest number of duplicates were analyzed. The overall duplicate rate was also plotted for each library. (PDF) [file pone.0214723.s003.pdf]

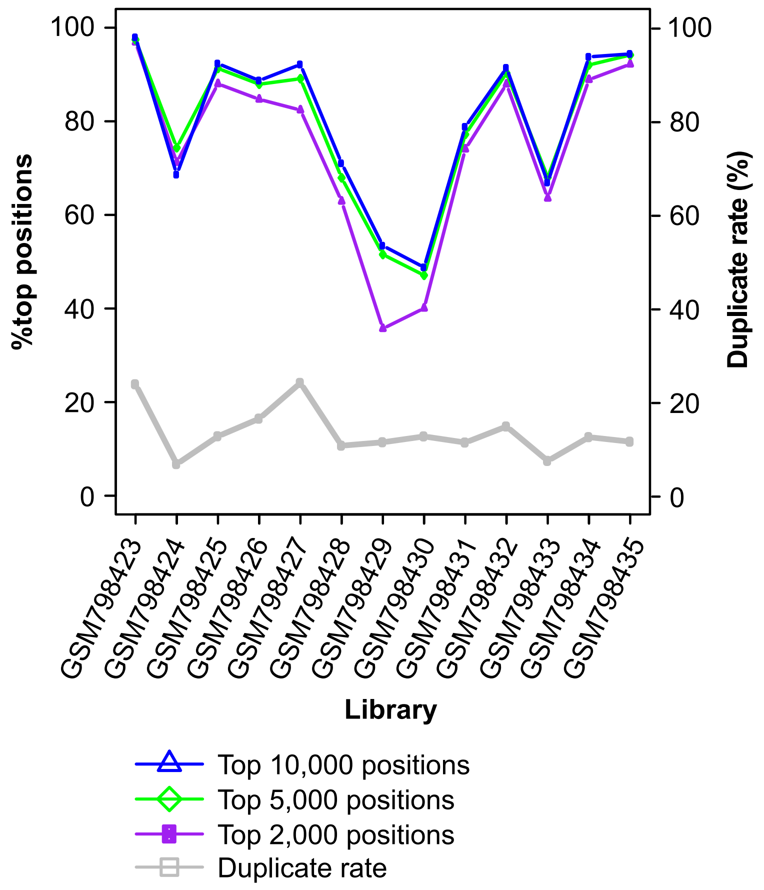

S3 Fig. A large proportion of top positions in the ER libraries are from peaks. For each library, the top 2,000, 5,000 and 20,000 positions with the highest number of duplicates were analyzed. The overall duplicate rate was also plotted for each library.
