# Supplementary material for: Identification of factors associated with duplicate rate in ChIP-seq data
Source: PLoS One. 2019 Apr 3;14(4):e0214723. doi: 10.1371/journal.pone.0214723 (PMC6447195; doi:10.1371/journal.pone.0214723)
Supplement: S4 Fig — The top 10,000 positions with the most duplicates from both ER peaks and non-peak regions were analyzed. Non-peak regions are those not covered by peaks. (PDF) [file pone.0214723.s004.pdf]

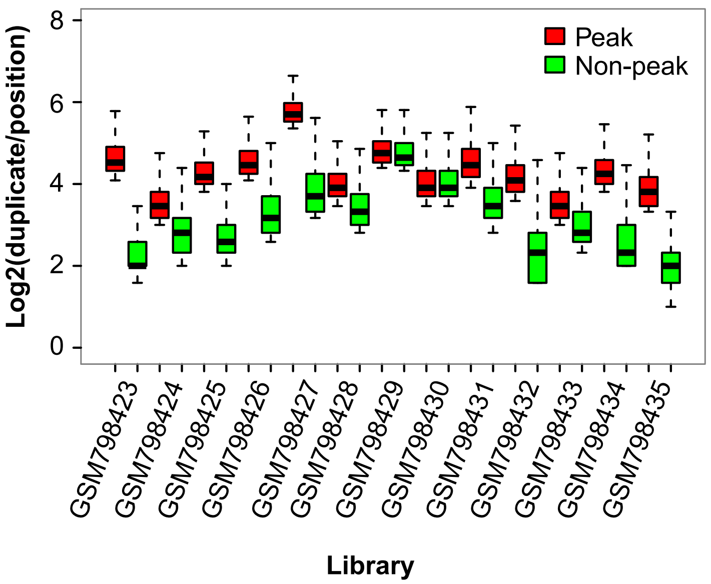

S4 Fig. Box plot of the number of duplicates per position. The top 10,000 positions with the most duplicates from both ER peaks and non-peak regions were analyzed. Non-peak regions are those not covered by peaks.
