# Supplementary material for: Identification of factors associated with duplicate rate in ChIP-seq data
Source: PLoS One. 2019 Apr 3;14(4):e0214723. doi: 10.1371/journal.pone.0214723 (PMC6447195; doi:10.1371/journal.pone.0214723)
Supplement: S5 Fig — Duplicate level was estimated as the number of reads per 10 million (RPK10M) on log2 scale. Breast cancer cell lines BT-474 (left) and TAM-R (right) were shown. See Fig 2 legend for details. (PDF) [file pone.0214723.s005.pdf]

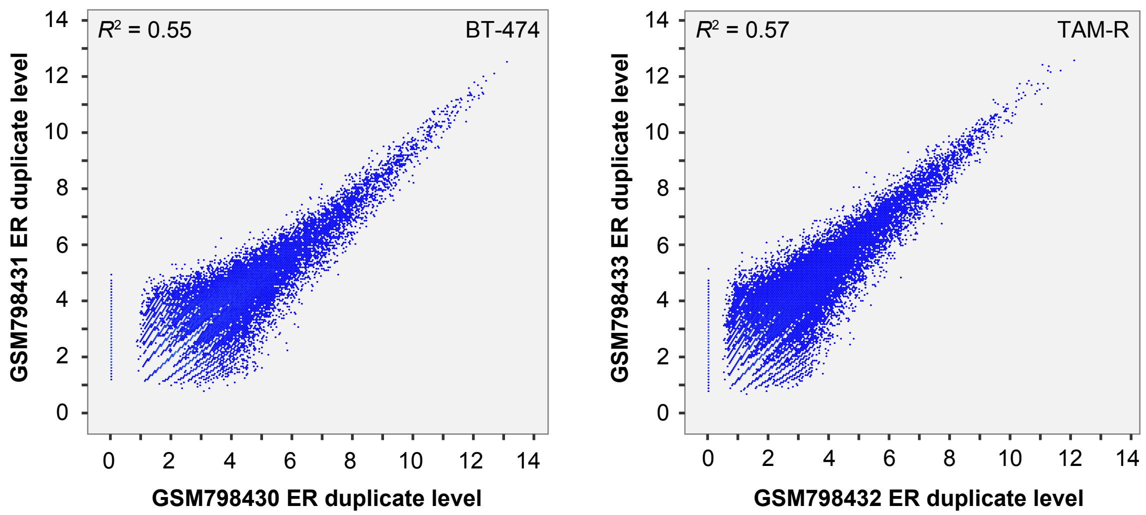

S5 Fig. Scatter plot of duplicate level within ER peaks between replicates. Duplicate level was estimated as the number of reads per 10 million (RPK10M) on log2 scale. Breast cancer cell lines BT-474 (left) and TAM-R (right) were shown. See Fig 2 legend for details.
