# Supplementary material for: Identification of factors associated with duplicate rate in ChIP-seq data
Source: PLoS One. 2019 Apr 3;14(4):e0214723. doi: 10.1371/journal.pone.0214723 (PMC6447195; doi:10.1371/journal.pone.0214723)
Supplement: S7 Fig — (A,C,E) Duplicate rate in 10 groups of peaks and in the corresponding regions in input. The dotted horizontal lines denote duplicate rates in the non-peak regions (> = 100 bp away from peaks). (B,D,F) Proportion of total library duplicates in each of the groups and in the corresponding regions in input. See Fig 3 legend for details. (PDF) [file pone.0214723.s007.pdf]

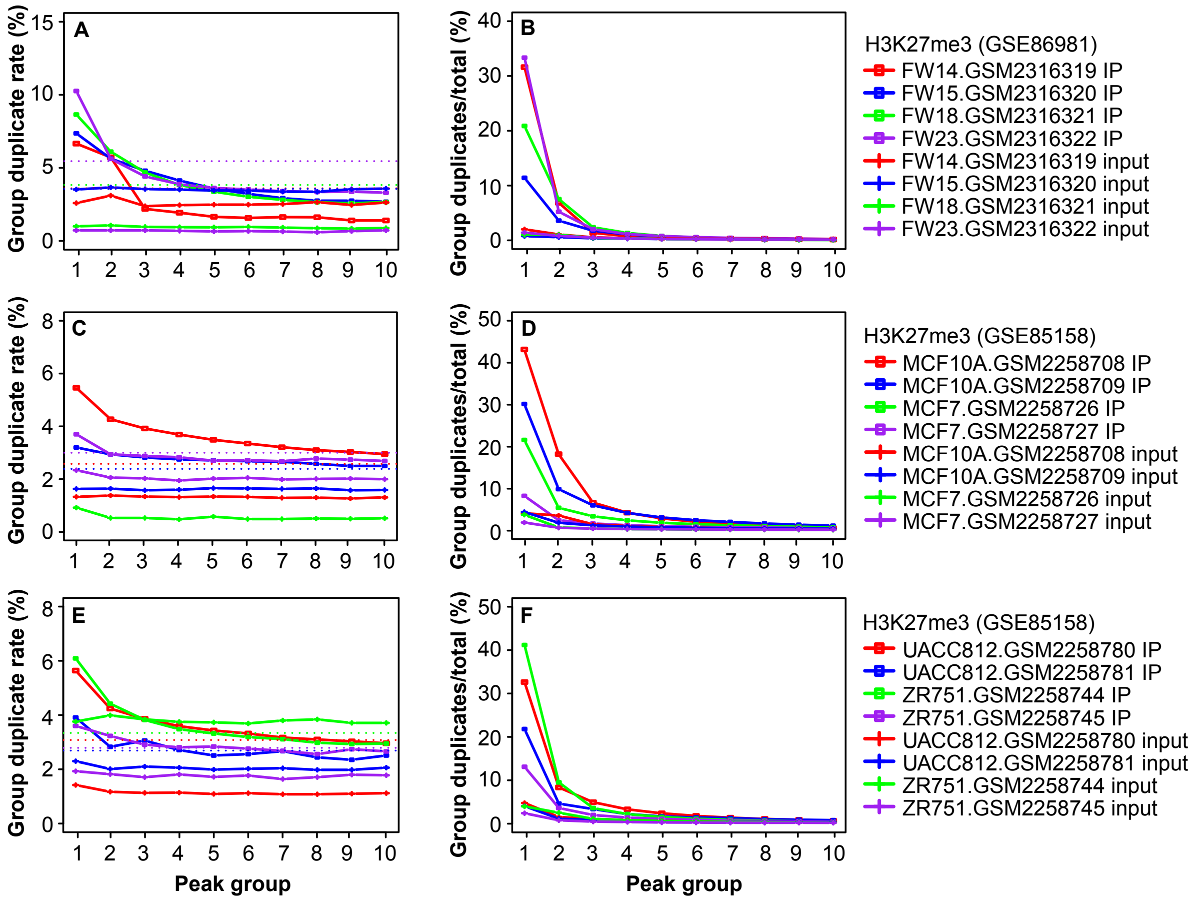

S7 Fig. Duplicate rate versus confidence level of H3K27me3 peaks. (**A,C,E**) Duplicate rate in 10 groups of peaks and in the corresponding regions in input. The dotted horizontal lines denote duplicate rates in the non-peak regions (>=100 bp away from peaks). (**B,D,F**) Proportion of total library duplicates in each of the groups and in the corresponding regions in input. See Figure 3 legend for details.
