# Supplementary material for: Identification of factors associated with duplicate rate in ChIP-seq data
Source: PLoS One. 2019 Apr 3;14(4):e0214723. doi: 10.1371/journal.pone.0214723 (PMC6447195; doi:10.1371/journal.pone.0214723)
Supplement: S8 Fig — Peaks were ranked (1 to 100) based on p value, with rank 1 indicating the top 1% of the peaks with the smallest p values. For each library, the top 10,000 positions with the most duplicates were identified from peaks, and the ranks of the peaks covering these positions were plotted. The top 10,000 positions were from relatively lower confident peaks in GSM798427 and GSM798429; they had over 2.5-fold more uniquely mapped reads than the other 11 libraries. (PDF) [file pone.0214723.s008.pdf]

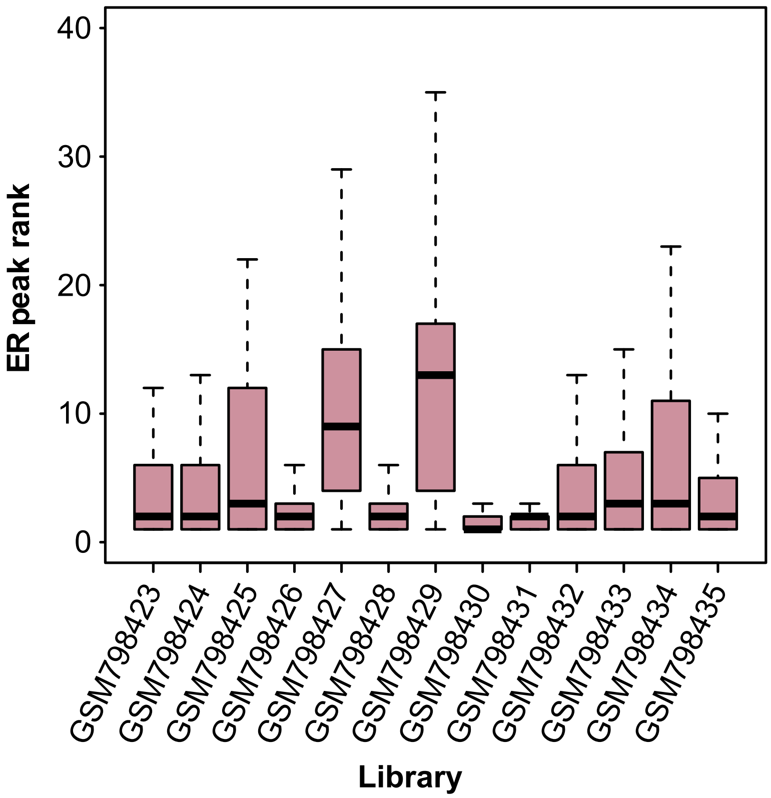

S8 Fig. Positions with the most duplicates tend to present in highly confident ER peaks. Peaks were ranked (1 to 100) based on *p* value, with rank 1 indicating the top 1% of the peaks with the smallest *p* values. For each library, the top 10,000 positions with the most duplicates were identified from peaks, and the ranks of the peaks covering these positions were plotted. The top 10,000 positions were from relatively lower confident peaks in GSM798427 and GSM798429; they had over 2.5-fold more uniquely mapped reads than the other 11 libraries.
