# Supplementary material for: Identification of factors associated with duplicate rate in ChIP-seq data
Source: PLoS One. 2019 Apr 3;14(4):e0214723. doi: 10.1371/journal.pone.0214723 (PMC6447195; doi:10.1371/journal.pone.0214723)
Supplement: S9 Fig — Peaks from group 1 (top 10% peaks with the lowest p values) and 5 (the fifth decile) were both split into 10 subgroups of equal size. Non-peak regions are 100 bp away from peaks. Flanking regions are peak 5’ and 3’ regions that are 300-bp away from the peaks and have the same size as the peaks. See S1 Table for sample information. (PDF) [file pone.0214723.s009.pdf]

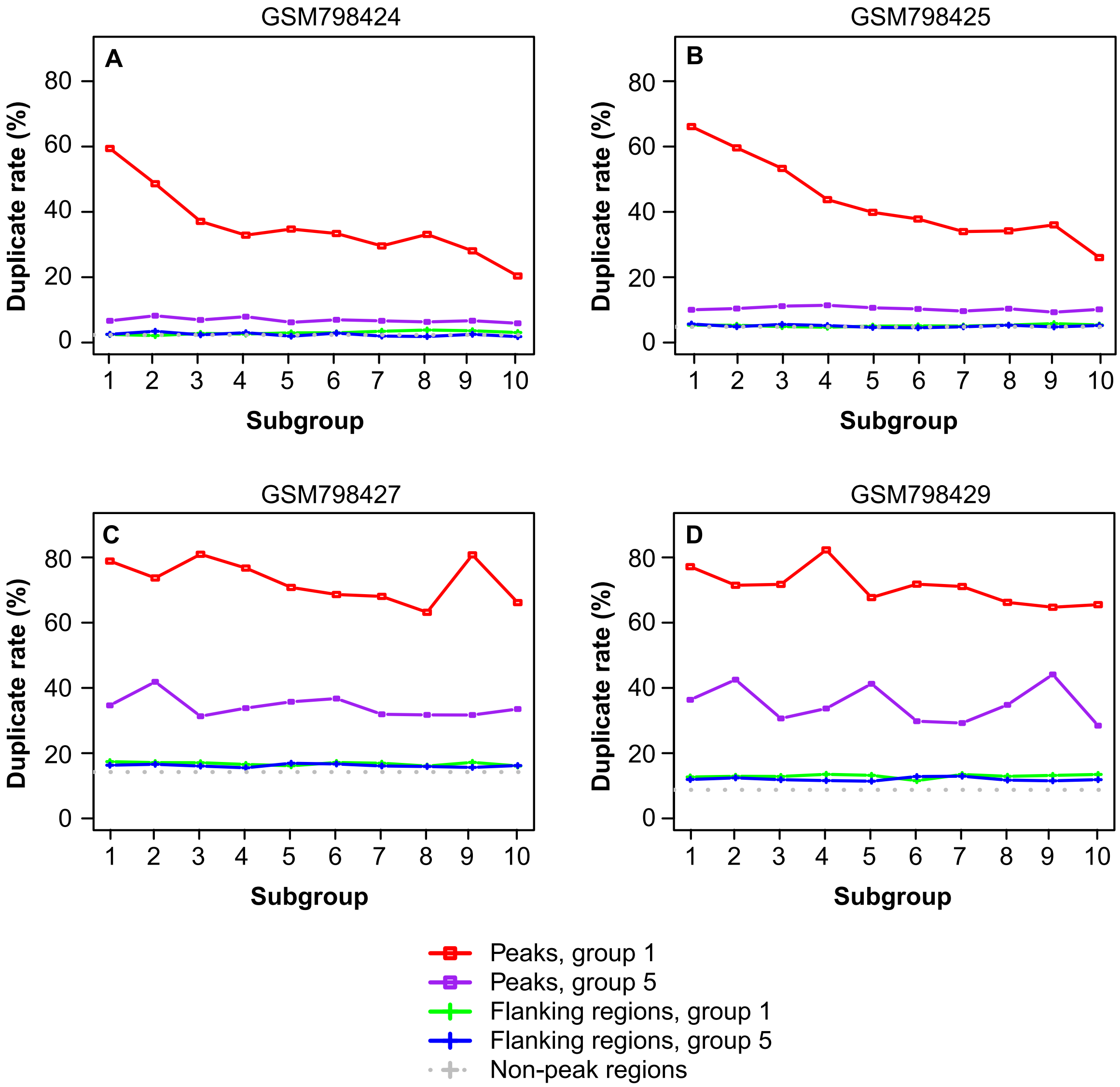

S9 Fig. Duplicate rate in ER peaks and the flanking regions. Peaks from group 1 (top 10% peaks with the lowest *p* values) and 5 (the fifth decile) were both split into 10 subgroups of equal size. Non-peak regions are 100 bp away from peaks. Flanking regions are peak 5' and 3' regions that are 300-bp away from the peaks and have the same size as the peaks. See S1 Table for sample information.
