# Supplementary material for: Identification of factors associated with duplicate rate in ChIP-seq data
Source: PLoS One. 2019 Apr 3;14(4):e0214723. doi: 10.1371/journal.pone.0214723 (PMC6447195; doi:10.1371/journal.pone.0214723)
Supplement: S10 Fig — Peaks were called with duplicate removal and the top 10% peaks with the smallest p values were shown. Number of raw reads in peaks was estimated from alignments both before and after duplicate removal. Y-axis indicates the input-subtracted number of reads per 10 million (RPK10M) on log2 scale. Only reads with a minimum mapping quality score of 20 and no mismatch at the first five bases were used. (PDF) [file pone.0214723.s010.pdf]

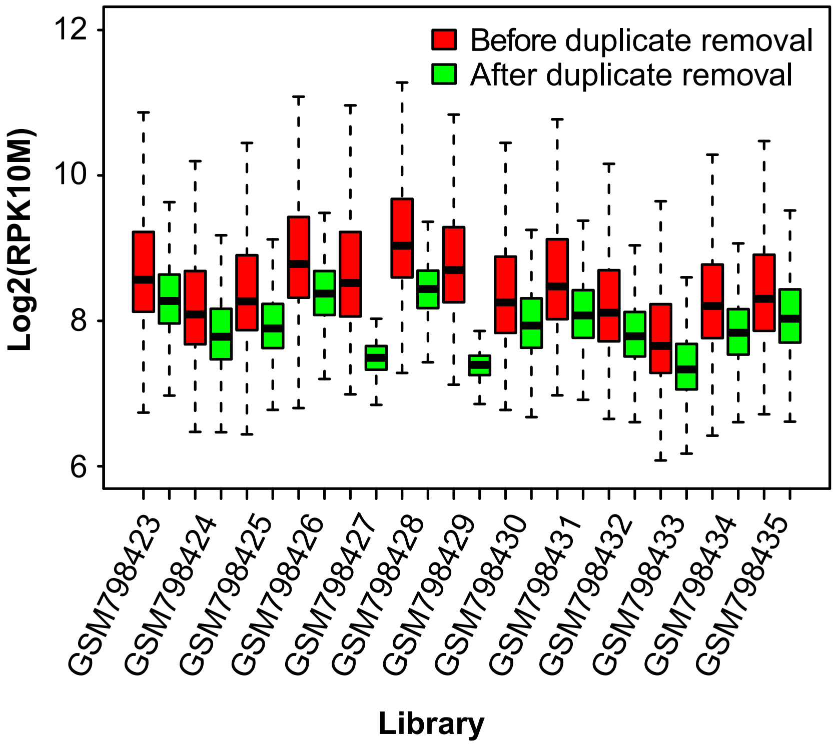

S10 Fig. Box plot of the number of reads in ER peaks with and without duplicate removal. Peaks were called with duplicate removal and the top 10% peaks with the smallest *p* values were shown. Number of raw reads in peaks was estimated from alignments both before and after duplicate removal. *Y*-axis indicates the input-subtracted number of reads per 10 million (RPK10M) on log2 scale. Only reads with a minimum mapping quality score of 20 and no mismatch at the first five bases were used.
