# Supplementary material for: Identification of factors associated with duplicate rate in ChIP-seq data
Source: PLoS One. 2019 Apr 3;14(4):e0214723. doi: 10.1371/journal.pone.0214723 (PMC6447195; doi:10.1371/journal.pone.0214723)
Supplement: S11 Fig — (A,B) H3K4me3 in lymphoblastoid cell lines. (C,D) H3K27me3 in fetal retinal tissue. (E,F) H3K27me3 in breast cancer cell lines. The curve was generated using the "lowess()" function in R. The coefficient of determination (R2) was calculated using Spearman rank coefficient. See Fig 4 legend for details. (PDF) [file pone.0214723.s011.pdf]

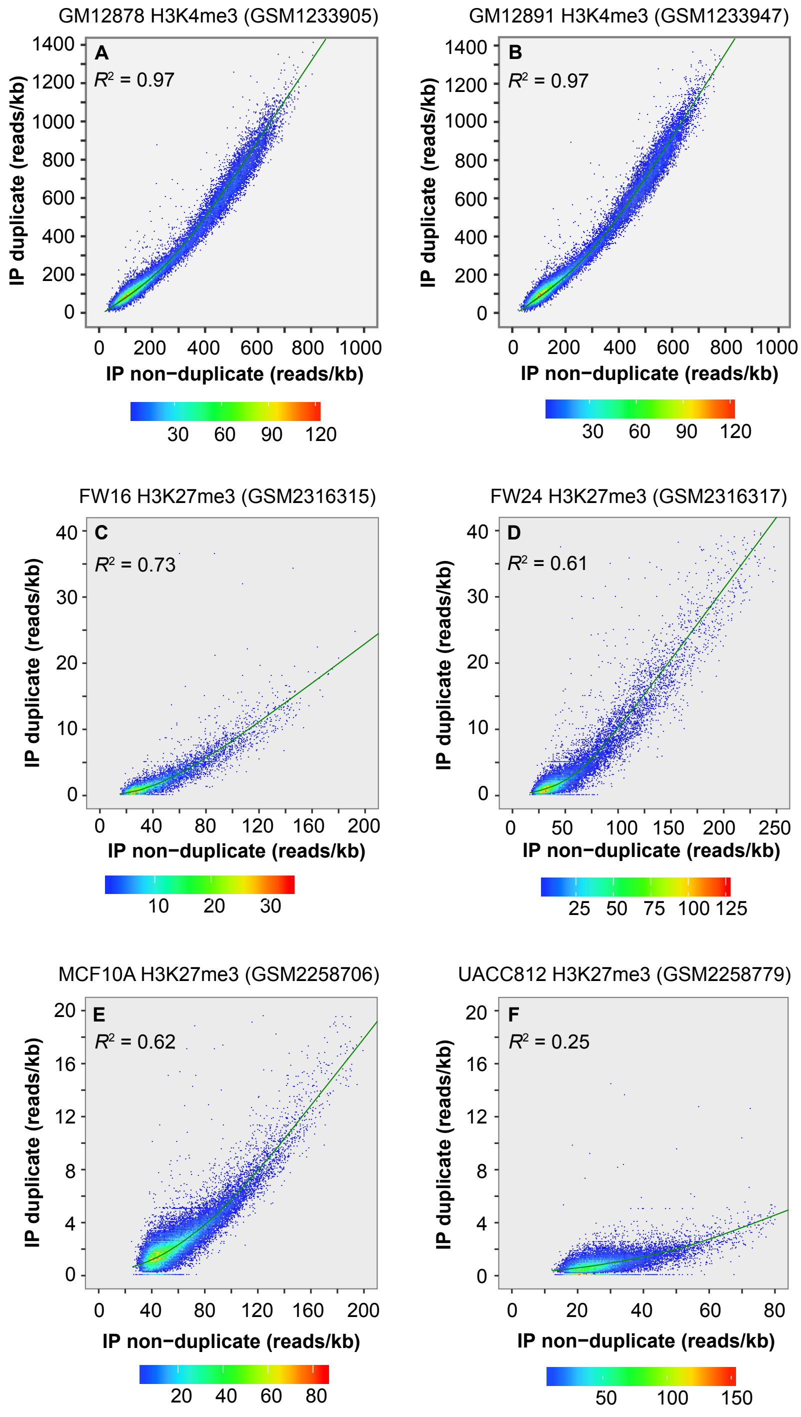

S11 Fig. Scatter plot of duplicate versus non-duplicate level within H3K4me3 and H3K27me3 peaks. (**A**-**B**) lymphoblastoid cell lines. (**C**-**D**) fetal retinal tissue. (**E**-**F**) breast cancer cell lines. The curve was generated using the "lowess()" function in R. R^2 value was calculated using Spearman rank correlation. See Fig 4 legend for details.
