# Supplementary material for: Identification of factors associated with duplicate rate in ChIP-seq data
Source: PLoS One. 2019 Apr 3;14(4):e0214723. doi: 10.1371/journal.pone.0214723 (PMC6447195; doi:10.1371/journal.pone.0214723)
Supplement: S12 Fig — The coefficient of determination (R2) was calculated using Spearman rank coefficient. See Fig 4 legend for details. (PDF) [file pone.0214723.s012.pdf]

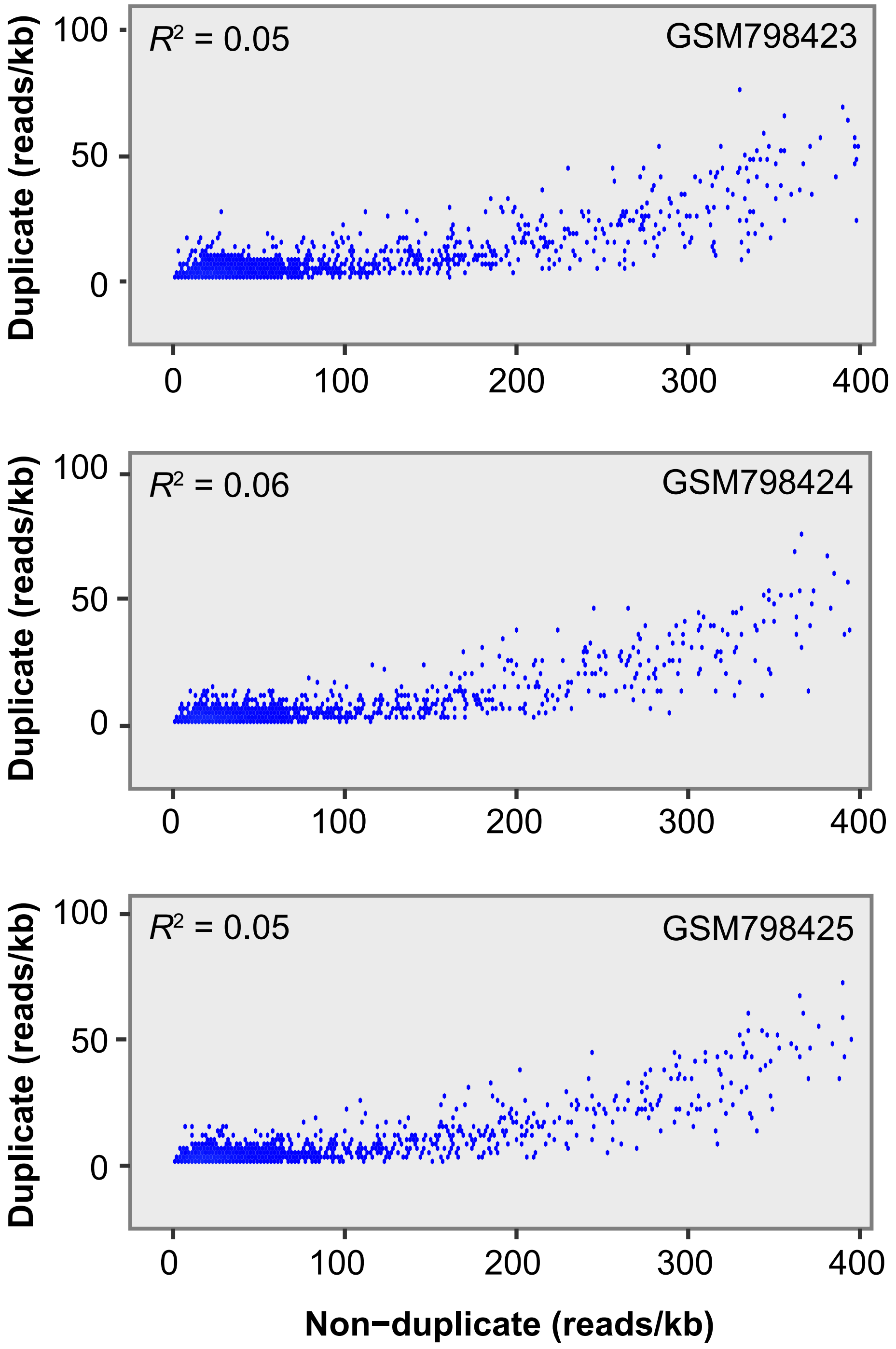

S12 Fig. Duplicate level versus non-duplicate level in ER peak corresponding regions in input. The coefficient of determination (R^2) was calculated using Spearman rank correlation coefficient. See Fig 4 legend for details.
