# Supplementary material for: Identification of factors associated with duplicate rate in ChIP-seq data
Source: PLoS One. 2019 Apr 3;14(4):e0214723. doi: 10.1371/journal.pone.0214723 (PMC6447195; doi:10.1371/journal.pone.0214723)
Supplement: S1 Table — (PDF) [file pone.0214723.s013.pdf]

S1 Table. Public ChIP-seq data used in this study

Accession	Sample	Mark	Rep	Len (bp)	Uniquely-mapped reads			Dup rate (%)	Ref
					Total	Non-duplicate	Duplicate		
GSM798423	MCF7	ER	1	36	21359033	16272592	5086441	23.81	(1)
GSM798424	MCF7	ER	2	36	21425134	19976300	1448834	6.76	(1)
GSM798425	MCF7	ER	3	36	27545925	24047021	3498904	12.7	(1)
GSM798426	ZR75	ER	1	36	22253226	18589932	3663294	16.46	(1)
GSM798427	ZR75	ER	2	36	79276870	60170275	19106595	24.1	(1)
GSM798428	T47D	ER	1	36	22081976	19720997	2360979	10.69	(1)
GSM798429	T47D	ER	2	36	78269473	69328093	8941380	11.42	(1)
GSM798430	BT474	ER	1	36	23114280	20186789	2927491	12.67	(1)
GSM798431	BT474	ER	2	36	23002429	20388556	2613873	11.36	(1)
GSM798432	TAMR	ER	1	36	24648235	21003432	3644803	14.79	(1)
GSM798433	TAMR	ER	2	36	31151281	28833101	2318180	7.44	(1)
GSM798434	MCF7	ER	1	36	27781866	24305683	3476183	12.51	(1)
GSM798435	MCF7	ER	2	36	18507910	16371761	2136149	11.54	(1)
GSM798440	MCF7	Input	1	36	21210223	20777470	432753	2.04	(1)
GSM798441	ZR75	Input	1	36	11502752	11231763	270989	2.36	(1)
GSM798442	T47D	Input	1	36	10705857	10518855	187002	1.75	(1)
GSM798443	BT474	Input	1	36	16793199	16112871	680328	4.05	(1)
GSM798444	TAMR	Input	1	36	31863425	29833676	2029749	6.37	(1)
GSM2574780	HepG2	NRF1	1	50	16387026	10519371	5867655	35.81	(2)
GSM2574769	K562	NRF1	1	50	20768242	15872708	4895534	23.57	(2)
GSM2574770	K562	NRF1	2	50	20925928	19089859	1836069	8.77	(2)
GSM2574771	K562	NRF1	3	50	19045016	15005621	4039395	21.21	(2)
GSM2574812	MCF7	NRF1	1	50	10347139	8499440	1847699	17.86	(2)
GSM2574813	MCF7	NRF1	2	50	10869614	10549815	319799	2.94	(2)
GSM2574832	HepG2	Input	1	50	35233595	34504410	729185	2.07	(2)
GSM2574827	K562	Input	1	50	41580911	40957796	623115	1.5	(2)
GSM2574824	MCF7	Input	1	50	26377261	25702061	675200	2.56	(2)
GSM1233880	GM10847	H3K4me3	1	101	34926147	27874383	7051764	20.19	(3)
GSM1233881	GM10847	H3K4me3	2	101	27058899	19971279	7087620	26.19	(3)
GSM1233905	GM12878	H3K4me3	1	101	49711836	22887937	26823899	53.96	(3)

GSM1233906	GM12878	H3K4me3	2	101	37401007	25153801	12247206	32.75	(3)
GSM1233907	GM12878	H3K4me3	3	101	38028364	23663127	14365237	37.78	(3)
GSM1233926	GM12890	H3K4me3	1	101	29593220	23354062	6239158	21.08	(3)
GSM1233927	GM12890	H3K4me3	2	101	35185060	24869723	10315337	29.32	(3)
GSM1233947	GM12891	H3K4me3	1	101	50129352	22368471	27760881	55.38	(3)
GSM1233948	GM12891	H3K4me3	2	101	33735359	23378283	10357076	30.7	(3)
GSM1233949	GM12891	H3K4me3	3	101	31866864	20731979	11134885	34.94	(3)
GSM1233969	GM12892	H3K4me3	1	101	47698522	22276584	25421938	53.3	(3)
GSM1233970	GM12892	H3K4me3	2	101	30027132	21221926	8805206	29.32	(3)
GSM1233971	GM12892	H3K4me3	3	101	37831639	22398153	15433486	40.8	(3)
GSM1233882	GM10847	Input	1	101	35316300	34282105	1034195	2.93	(3)
GSM1233908	GM12878	Input	1	101	47185858	34585263	12600595	26.7	(3)
GSM1233928	GM12890	Input	1	101	31840943	30157510	1683433	5.29	(3)
GSM1233950	GM12891	Input	1	101	51285404	40353756	10931648	21.32	(3)
GSM1233972	GM12892	Input	1	101	55463643	46008701	9454942	17.05	(3)
SRR2992404	HeLa	H3K4me3	1	51	140449480	86837367	53612113	38.17	(4)
SRR2992405	HeLa	H3K4me3	2	51	143455408	88485544	54969864	38.32	(4)
SRR2992406	HeLa	H3K4me3	3	51	160077186	95778707	64298479	40.17	(4)
GSM2316314	FW14	H3K27me3	1	51	22930433	21981537	948896	4.14	(5)
GSM2316315	FW16	H3K27me3	1	50	26494214	25473343	1020871	3.85	(5)
GSM2316316	FW18	H3K27me3	1	51	34874087	33317054	1557033	4.46	(5)
GSM2316317	FW24	H3K27me3	1	50	34045087	31904345	2140742	6.29	(5)
GSM2316319	FW14	H3K36me3	1	51	25148476	24667598	480878	1.91	(5)
GSM2316320	FW15	H3K36me3	1	50	22577220	20477412	2099808	9.3	(5)
GSM2316321	FW18	H3K36me3	1	51	34869030	33524232	1344798	3.86	(5)
GSM2316322	FW23	H3K36me3	1	50	25276028	24522202	753826	2.98	(5)
GSM2316360	FW14	Input	1	50	29836124	28954648	881476	2.95	(5)
GSM2316361	FW15	Input	1	50	23301393	22792378	509015	2.18	(5)
GSM2316362	FW16	Input	1	50	27324867	26321251	1003616	3.67	(5)
GSM2316363	FW18	Input	1	51	25179150	24801280	377870	1.5	(5)
GSM2316365	FW23	Input	1	51	17684285	17436314	247971	1.4	(5)
GSM2316366	FW24	Input	1	50	15760414	15553359	207055	1.31	(5)
GSM2258688	76NF2V	H3K27me3	1	36	50053973	47998676	2055297	4.11	(6)
GSM2258689	76NF2V	H3K27me3	2	36	31270025	30269337	1000688	3.2	(6)

GSM2258760	MB361	H3K27me3	1	36	57364806	54944570	2420236	4.22	(6)
GSM2258761	MB361	H3K27me3	2	36	32442483	31483899	958584	2.95	(6)
GSM2258706	MCF10A	H3K27me3	1	36	51669072	49600697	2068375	4	(6)
GSM2258707	MCF10A	H3K27me3	2	36	23093796	22466317	627479	2.72	(6)
GSM2258724	MCF7	H3K27me3	1	36	44335211	28363763	15971448	36.02	(6)
GSM2258725	MCF7	H3K27me3	2	36	26917903	26102451	815452	3.03	(6)
GSM2258778	UACC812	H3K27me3	1	36	58758376	56526845	2231531	3.8	(6)
GSM2258779	UACC812	H3K27me3	2	36	24409333	23704717	704616	2.89	(6)
GSM2258742	ZR751	H3K27me3	1	36	43453702	41615247	1838455	4.23	(6)
GSM2258743	ZR751	H3K27me3	2	36	23720248	23035418	684830	2.89	(6)
GSM2258690	76NF2V	H3K36me3	1	36	47440920	45878037	1562883	3.29	(6)
GSM2258691	76NF2V	H3K36me3	2	36	32033764	30565279	1468485	4.58	(6)
GSM2258762	MB361	H3K36me3	1	36	45887840	44099328	1788512	3.9	(6)
GSM2258763	MB361	H3K36me3	2	36	23128814	22394079	734735	3.18	(6)
GSM2258708	MCF10A	H3K36me3	1	36	40093983	38787401	1306582	3.26	(6)
GSM2258709	MCF10A	H3K36me3	2	36	24677639	23722610	955029	3.87	(6)
GSM2258726	MCF7	H3K36me3	1	36	65076310	58566177	6510133	10	(6)
GSM2258727	MCF7	H3K36me3	2	36	24321210	23118939	1202271	4.94	(6)
GSM2258780	UACC812	H3K36me3	1	36	61536555	58450481	3086074	5.02	(6)
GSM2258781	UACC812	H3K36me3	2	36	16769008	16219844	549164	3.27	(6)
GSM2258744	ZR751	H3K36me3	1	36	47687434	45970765	1716669	3.6	(6)
GSM2258745	ZR751	H3K36me3	2	36	23278359	22618297	660062	2.84	(6)
GSM2258702	76NF2V	Input	1	36	35766057	35261677	504380	1.41	(6)
GSM2258703	76NF2V	Input	2	36	20795614	20364387	431227	2.07	(6)
GSM2258774	MB361	Input	1	36	38128273	37613495	514778	1.35	(6)
GSM2258775	MB361	Input	2	36	21872989	21363210	509779	2.33	(6)
GSM2258720	MCF10A	Input	1	36	61744537	60612759	1131778	1.83	(6)
GSM2258721	MCF10A	Input	2	36	15918399	15597818	320581	2.01	(6)
GSM2258738	MCF7	Input	1	36	23659654	23411185	248469	1.05	(6)
GSM2258739	MCF7	Input	2	36	21885042	21306567	578475	2.64	(6)
GSM2258792	UACC812	Input	1	36	48384203	47570218	813985	1.68	(6)
GSM2258793	UACC812	Input	2	36	22451322	21911029	540293	2.41	(6)
GSM2258756	ZR751	Input	1	36	44018883	42117200	1901683	4.32	(6)
GSM2258757	ZR751	Input	2	36	19923712	19515963	407749	2.05	(6)

ER ChIP-seq data in five breast cancer cell lines were downloaded from NCBI under accession GSE32222. NRF1 ChIP-seq data were from GSE97661. H3K4me3 ChIP-seq data in five lymphoblastoid cell lines were from GSE50893. PCR-free H3K4me3 ChIP-seq data in HeLa were from SRP067250. H3K27me3 and H3K36me3 ChIP-seq data in fetal retinal tissue (FW14, FW15, FW16, FW18, FW23 and FW24) were from GSE86981. H3K27me3 and H3K36me3 ChIP-seq data in six breast cancer cell lines (76NF2V, MB361, MCF10A, MCF7, UACC812, and ZR751) were from GSE85158. Only uniquely mapped reads with a minimum mapping quality score of 20 and no mismatch at the first five bases were used. Dup rate, duplicate rate; Rep, replicate; Len, length.

References

1. Ross-Innes CS, Stark R, Teschendorff AE, Holmes KA, Ali HR, Dunning MJ, et al. Differential oestrogen receptor binding is associated with clinical outcome in breast cancer. Nature. 2012; 481(7381):389-93.
2. Venkataraman A, Yang K, Irizarry J, Mackiewicz M, Mita P, Kuang Z, et al. A toolbox of immunoprecipitation-grade monoclonal antibodies to human transcription factors. Nature methods. 2018; 15(5):330-8.
3. Kasowski M, Kyriazopoulou-Panagiotopoulou S, Grubert F, Zaugg JB, Kundaje A, Liu Y, et al. Extensive variation in chromatin states across humans. Science (New York, NY). 2013; 342(6159):750-2.
4. Sundaram AY, Hughes T, Biondi S, Bolduc N, Bowman SK, Camilli A, et al. A comparative study of ChIP-seq sequencing library preparation methods. BMC genomics. 2016; 17(1):816.
5. Aldiri I, Xu B, Wang L, Chen X, Hiler D, Griffiths L, et al. The Dynamic Epigenetic Landscape of the Retina During Development, Reprogramming, and Tumorigenesis. Neuron. 2017; 94(3):550-68.e10.
6. Franco HL, Nagari A, Malladi VS, Li W, Xi Y, Richardson D, et al. Enhancer transcription reveals subtype-specific gene expression programs controlling breast cancer pathogenesis. Genome research. 2018; 28(2):159-70.
