# Supplementary material for: Identification of factors associated with duplicate rate in ChIP-seq data
Source: PLoS One. 2019 Apr 3;14(4):e0214723. doi: 10.1371/journal.pone.0214723 (PMC6447195; doi:10.1371/journal.pone.0214723)
Supplement: S2 Table — (PDF) [file pone.0214723.s014.pdf]

S2 Table. Number of ER peaks with the most highly duplicated positions

Accession	Sample	Dup rate (%)	Number of top positions*			Peaks w/ ER motif (%)**
			2000	5000	10000	
GSM798423	MCF7	23.81	73	189	328	73.48
GSM798424	MCF7	6.76	91	223	408	72.55
GSM798425	MCF7	12.70	68	164	295	77.63
GSM798426	ZR75	16.46	98	188	326	87.12
GSM798427	ZR75	24.10	86	146	236	79.24
GSM798428	T47D	10.69	80	161	263	77.95
GSM798429	T47D	11.42	67	120	196	83.16
GSM798430	BT474	12.67	68	132	240	86.67
GSM798431	BT474	11.36	53	116	198	86.36
GSM798432	TAMR	14.79	62	177	363	78.79
GSM798433	TAMR	7.44	57	182	396	78.28
GSM798434	MCF7	12.51	63	144	284	73.94
GSM798435	MCF7	11.54	79	196	372	71.77

ER peaks were identified using MACS after duplicate removal.

* The top 2,000, 5,000, and 10,000 positions were those that had the highest number of duplicates in peaks.

** ER motif was identified from peaks with the top 10,000 positions.
