# Supplementary material for: Identification of factors associated with duplicate rate in ChIP-seq data
Source: PLoS One. 2019 Apr 3;14(4):e0214723. doi: 10.1371/journal.pone.0214723 (PMC6447195; doi:10.1371/journal.pone.0214723)
Supplement: S3 Table — (PDF) [file pone.0214723.s015.pdf]

S3 Table. Number of ER peaks called with and without duplicate removal

Accession	Dup rate (%)	Dup in peak (%)	Peaks w/ duplicate removal			Peaks w/o duplicate removal			Caller
			Shared	Unique	Unique w/ motif	Shared	Unique	Unique w/ motif	
GSM798423	23.81	60.98	60789	7152	2215 (30.97)	61011	1595	732 (45.89)	macs2
GSM798424	6.76	71.25	39977	5166	1411 (27.31)	40092	181	29 (16.02)	macs2
GSM798425	12.7	71.79	52740	3378	1216 (36)	52810	756	218 (28.84)	macs2
GSM798426	16.46	80.68	49341	4615	435 (9.43)	49515	316	27 (8.54)	macs2
GSM798427	24.1	53.26	31632	2604	375 (14.4)	31578	775	155 (20)	macs2
GSM798428	10.69	47.63	15315	542	53 (9.78)	15281	551	88 (15.97)	macs2
GSM798429	11.42	26.89	6563	37	0 (0)	6491	892	319 (35.76)	macs2
GSM798430	12.67	39.7	30361	1291	183 (14.18)	30321	1369	111 (8.11)	macs2
GSM798431	11.36	59.37	30354	1594	153 (9.6)	30336	848	88 (10.38)	macs2
GSM798432	14.79	55.12	55963	923	114 (12.35)	55808	4714	950 (20.15)	macs2
GSM798433	7.44	48.98	43255	888	162 (18.24)	43147	2502	611 (24.42)	macs2
GSM798434	12.51	73.99	56217	3573	964 (26.98)	56269	796	344 (43.22)	macs2
GSM798435	11.54	67.71	54883	2224	374 (16.82)	54858	2399	654 (27.26)	macs2
GSM798423	23.81	44.76	67579	5676	2011 (35.43)	67579	3889	1449 (37.26)	findPeaks
GSM798424	6.76	28.06	48317	3565	1464 (41.07)	48317	620	195 (31.45)	findPeaks
GSM798425	12.7	36.32	63540	4123	1458 (35.36)	63540	1430	390 (27.27)	findPeaks
GSM798426	16.46	39.8	62723	5840	1907 (32.65)	62723	834	194 (23.26)	findPeaks
GSM798427	24.1	58.62	44420	3801	1389 (36.54)	44420	1017	242 (23.80)	findPeaks
GSM798428	10.69	41.13	22688	1149	419 (36.47)	22688	1215	251 (20.66)	findPeaks
GSM798429	11.42	56.56	10921	508	194 (38.19)	10921	769	184 (23.93)	findPeaks
GSM798430	12.67	34.79	39786	2035	619 (30.42)	39786	2369	749 (31.62)	findPeaks
GSM798431	11.36	39.07	39099	2398	769 (32.07)	39099	1311	396 (30.21)	findPeaks
GSM798432	14.79	36.87	56755	2326	643 (27.64)	56755	3028	733 (24.21)	findPeaks
GSM798433	7.44	29.01	45117	2057	553 (26.88)	45117	1305	276 (21.15)	findPeaks
GSM798434	12.51	34.88	67433	4529	1147 (25.33)	67433	1497	294 (19.64)	findPeaks
GSM798435	11.54	31.9	57429	3053	991 (32.46)	57429	1871	509 (27.20)	findPeaks

Number in the parenthesis refers to the percentage of unique peaks with ER motif. For unique peaks identified by findPeaks in ZR75 (GSM798426-GSM798427), T47D (GSM798428-GSM798429) and BT474 (GSM798430-GSM798431), match to AP-2 motif was also counted as AP-2 motif was commonly adjacent to ER motif (PMID: 21179027). %Dup in peak, the number of duplicates in peaks over the total duplicates in the library; Dup, duplicate.
