# Supplementary material for: Identification of factors associated with duplicate rate in ChIP-seq data
Source: PLoS One. 2019 Apr 3;14(4):e0214723. doi: 10.1371/journal.pone.0214723 (PMC6447195; doi:10.1371/journal.pone.0214723)
Supplement: S4 Table — (PDF) [file pone.0214723.s016.pdf]

S4 Table. Number of NRF1 peaks called with and without duplicate removal

Accession	Sample	Rep	Dup rate (%)	Dup in peak (%)	With Duplicate removal		No duplicate removal	
					Shared	Unique	Shared	Unique
GSM2574780	HepG2	1	35.81	48.34	7680	147 (59.18;50.34;67.35)	7676	1528 (23.89;22.25;25.72)
GSM2574769	K562	1	23.57	81.38	15078	1272 (12.89;57.55;71.23)	15153	115 (36.52;40;44.35)
GSM2574770	K562	2	8.77	30.06	7144	80 (na;68.75;80)	7138	355 (51.55;60.85;71.27)
GSM2574771	K562	3	21.21	79.49	13177	999 (36.84;62.26;76.68)	13234	185 (57.3;35.68;31.89)
GSM2574812	MCF7	1	17.86	91.61	8321	589 (20.03;64.69;60.1)	8338	32 (na;53.13;56.25)
GSM2574813	MCF7	2	2.94	29.08	3907	18 (na;72.22;72.22)	3906	127 (58.27;77.95;77.95)

Number in the parenthesis refers to the percentage of unique peaks with the NRF1 DNA binding motif, overlapping the 4-kb windows centered on the transcriptional start sites and the H3K4me3 peaks from the reference epigenome in HepG2, K562 and HMEC (PMID: 25693563). Rep, replicate; Dup, duplicate; na, not available due to small peak number (<100).
