# Supplementary material for: Identification of factors associated with duplicate rate in ChIP-seq data
Source: PLoS One. 2019 Apr 3;14(4):e0214723. doi: 10.1371/journal.pone.0214723 (PMC6447195; doi:10.1371/journal.pone.0214723)
Supplement: S5 Table — (PDF) [file pone.0214723.s017.pdf]

S5 Table. Number of H3K4me3 peaks called with and without duplicate removal

Accession	Sample	Rep	Dup rate (%)	Dup in peak (%)	With Duplicate removal		No duplicate removal		Caller
					Shared	Unique	Shared	Unique	
GSM1233880	GM10847	1	20.19	95.67	32286	3379 (26.63;54.87)	33595	100 (32.26;34)	macs2
GSM1233881	GM10847	2	26.19	96.73	35411	3857 (23.56;51.36)	36892	124 (22.94;41.94)	macs2
GSM1233905	GM12878	1	53.96	82.11	33262	837 (20.2;63.32)	32814	4762 (23.22;53.84)	macs2
GSM1233906	GM12878	2	32.75	93.67	29710	2805 (22.55;65.31)	30943	420 (16.67;49.52)	macs2
GSM1233907	GM12878	3	37.78	91.23	33511	2225 (21.56;61.39)	34422	1096 (19.39;51.55)	macs2
GSM1233926	GM12890	1	21.08	94.02	29498	2754 (21.07;60.02)	30549	138 (27.45;48.55)	macs2
GSM1233927	GM12890	2	29.32	97.9	25014	4960 (20.75;64.31)	26930	12 (22.45;25)	macs2
GSM1233947	GM12891	1	55.38	83.71	33598	386 (19.2;56.74)	32775	5793 (19.96;48.44)	macs2
GSM1233948	GM12891	2	30.7	94.2	28687	2383 (22.44;61.39)	29634	277 (21.72;48.01)	macs2
GSM1233949	GM12891	3	34.94	89.5	36415	1385 (21.65;49.82)	36804	2123 (23.91;43.1)	macs2
GSM1233969	GM12892	1	53.3	86.73	32086	658 (24.85;56.08)	31828	3900 (19.86;48.62)	macs2
GSM1233970	GM12892	2	29.32	93.62	29270	2209 (24.96;58.22)	30168	437 (37.5;42.33)	macs2
GSM1233971	GM12892	3	40.8	88.49	37466	1274 (20.77;50.16)	37693	2856 (19.92;41.98)	macs2
GSM1233880	GM10847	1	20.19	95.7	27019	1559 (14.18;46.57)	27212	94 (14.89;39.36)	findPeaks
GSM1233881	GM10847	2	26.19	96.74	27592	1525 (15.54;45.64)	27808	131 (18.32;34.35)	findPeaks
GSM1233905	GM12878	1	53.96	83.95	27472	1784 (11.88;66.7)	26797	5532 (14.46;29.39)	findPeaks
GSM1233906	GM12878	2	32.75	94.5	27199	2094 (12.51;61.22)	27321	933 (12;43.94)	findPeaks
GSM1233907	GM12878	3	37.78	92.48	28752	1959 (12.05;59.78)	28793	1447 (11.68;34.28)	findPeaks
GSM1233926	GM12890	1	21.08	94.52	25757	1878 (13.31;50.16)	25930	409 (9.05;51.34)	findPeaks
GSM1233927	GM12890	2	29.32	98.11	23066	2752 (13.59;58.47)	23323	214 (11.68;66.36)	findPeaks
GSM1233947	GM12891	1	55.38	85.23	29464	1663 (10.22;57.97)	28926	5990 (12.8;25.16)	findPeaks
GSM1233948	GM12891	2	30.7	94.74	27005	1620 (14.88;55.37)	27103	669 (11.51;44.1)	findPeaks
GSM1233949	GM12891	3	34.94	90.89	31739	1215 (11.19;45.76)	31633	2154 (13.32;27.11)	findPeaks
GSM1233969	GM12892	1	53.3	87.89	27630	1341 (11.33;63.68)	27306	3953 (13.89;32.1)	findPeaks
GSM1233970	GM12892	2	29.32	94.23	26696	1494 (14.46;50.33)	26800	628 (12.9;42.36)	findPeaks
GSM1233971	GM12892	3	40.8	89.7	31736	1032 (10.66;52.03)	31470	3335 (12.77;26.36)	findPeaks

Number in the parenthesis refers to the percentage of unique peaks overlapping the 4-kb windows centered on the transcriptional start sites and H3K4me3 peaks from the GM12878 reference epigenome (PMID: 25693563). Rep, replicate; Dup, duplicate.
